# Selection of Reference Genes for Expression Normalization by RT-qPCR in *Dracocephalum moldavica* L.

**DOI:** 10.3390/cimb46060375

**Published:** 2024-06-20

**Authors:** Shasha Li, Xiaomin Ge, Guoqing Bai, Chen Chen

**Affiliations:** Shaanxi Engineering Research Centre for Conservation and Utilization of Botanical Resources, Xi’an Botanical Garden of Shaanxi Province, Institute of Botany of Shaanxi Province, No. 17 Cuihua South Road, Xi’an 710061, China; lishasha@xab.ac.cn (S.L.); gxm@xab.ac.cn (X.G.); bgq@ms.xab.ac.cn (G.B.)

**Keywords:** *Dracocephalum moldavica*, drought stress, gene expression, reference gene, RT-qPCR

## Abstract

*Dracocephalum moldavica* is widely used as an ornamental, medicine, and perfume in industry. Real-time fluorescence quantitative polymerase chain reaction (RT-qPCR) is widely and accurately utilized for gene expression evaluations. Selecting optimal reference genes is essential for normalizing RT-qPCR results. However, the identification of suitable reference genes in *D*. *moldavica* has not been documented. A total of 12 reference genes in *D*. *moldavica* were identified by PEG6000 (15%) treatment under hypertonia conditions in different tissues (roots, stem, leaves, flower, seeds and sepal) and during three stages of flower development, then used to validate the expression stability. There were four algorithms (delta Ct, geNorm, NormFinder, and BestKeeper) used to analyze the stability. Finally, the RefFinder program was employed to evaluate the candidate reference genes’ stability. The results showed that *ACTIN*, *glyceraldehyde-3-phosphate dehydrogenase* (*GAPDH*), and *EF1α* (*elongation factor-1α*) were stable reference genes under the PEG6000 treatment. *Heat shock protein 70* (*HSP70*) was the most stable gene across different flower development stages. *ADP-ribosylation factor* (*ARF*) was the most stable gene in different tissues and total samples. This study provides reliable gene expression studies for future research in *D*. *moldavica*.

## 1. Introduction

*Dracocephalum moldavica* L. is an annual herb from the *Lamiaceae* family which is widely distributed in regions including Northwest China, Russia, Asia, Northern Europe, and North America [1]. The genus’s resistance to drought and adaptability is influenced by its environment, which is warm and arid [2]. The entire herb of *D. moldavica* possesses medicinal properties. Its taste is characterized by a bitter and cool flavor, and it is known for heat-clearing and dampness-relieving effects [3]. The essential oil extracted from *D. moldavica* contains various compounds, such as flavonoids, lignans, caffeic acid, terpenoids, and phenylpropanoids, which play essential roles in the plant’s defense system and have applications in the medicinal and food industries [4,5,6]. The active constituents of *D. moldavica* demonstrate sedative and analgesic properties [3], as well as effectiveness in soothing the throat and alleviating cough [6]. Furthermore, the visually appealing light lavender flowers and attractive shape of *D. moldavica* make it suitable for horticultural and landscaping purposes. Current research on *D. moldavica* primarily focuses on analyzing its medicinal components and their pharmacological effects [1,4,7]. There is a scarcity of studies exploring the molecular mechanisms underlying the biosynthesis pathway of important components in volatile oils or the formation of drought tolerance due to insufficient genetic information obtained through DNA or RNA sequencing. Moreover, there is a scarcity of reported studies on the stabilization of genes for evaluating the gene expression level in *D. moldavica*.

Real-time fluorescence quantitative polymerase chain reaction (RT-qPCR) is a crucial technology utilized for the detection of gene expression [8]. Its widespread application in molecular biology experiments is attributed to its high sensitivity and reliable repeatability. The accuracy of RT-qPCR is influenced by various factors, including RNA quality, primer amplification efficiency and specificity, and the stability of reference genes. The appropriate selection of reference genes is foundational for successfully executing an RT-qPCR experiment [9,10,11]. Theoretically, an ideal reference gene should exhibit stable expression across tissues and under various biological and abiotic conditions. However, there is no definitive evidence of genes that are consistently expressed stably in all test samples and all species. For instance, under drought stress, *GAPDH* has been found to be a suitable reference gene in roots of *Avena sativa* L. [12], but it exhibits less stability in peach [13]. Other studies have reported that *GAPDH* is most stable at different developmental stages of purple-and white flowers in *Allium wallichii* [14]. Therefore, each plant has its own more appropriate reference genes. Numerous studies have been conducted to screen the stability of reference genes in many plant species, including *Schima superba* [11], *Ipomoea batatas* [9], *Stipagrostis pennata* [10], *Schima superba* [15], *Avena sativa* [12], *Vitis vinifera* [16], *Populus* [17], *Solanum nigrum* [18], *Cryptomeria fortune* [19], *Stellera chamaejasme* [20], *Dianthus broteri* [21], *Pyrus pyrifolia* [22], *Arachis hypogaea* [23], and *Nitraria tangutorum* [24]. In plant gene expression studies, reference genes such as *elongation factor-1α* (*EF1α*), *18S rRNA*, *glyceraldehyde-3-phosphate dehydrogenase* (*GAPDH*), *Actin* (*ACT*), *S-adenosylmethionine synthase* (*SAMS*), and *tubulin* (*TUB*) have commonly been employed for optimizing gene expression [9,25,26,27].

To date, there has been a lack of comprehensive research on identifying a stable reference gene for *D. moldavica*. Utilizing our transcriptome data obtained from leaves and roots under PEG6000 treatment of *D. moldavica*, we identified 12 candidate reference genes, namely, *18S rRNA* [19], *ACT* [15,19], *HIS4* (*Histone 4*) [28], *HSP70* (*Heat shock protein 70*) [29], *28S rRNA* [30], *ARF* [31], *EF1α* [24], *SAMDC* (*S-adenosylmethionine decarboxylase*) [21], *GAPDH* [10], *eIF4α* (*Eukaryotic initiation factor 4-α*) [10], *TUB* [32], and *CAC* (*Clathrin adaptor complex*) [33], to evaluate the most suitable reference genes of *D. moldavica*. The stabilities of the expression levels of these 12 candidate reference genes were evaluated in various RNA samples, such as leaves and roots, under 15% PEG6000 treatments, in different organs (roots, stem, leaves, flower, seeds and sepal), and at three stages of flower development (bud, initial flowering, and full flowering stages, respectively). Four statistical methods (delta Ct, geNorm, Normfinder, and BestKeeper) were used to identify the most stable reference genes. Then, the RefFinder program was utilized to comprehensively evaluate and screen the most stable reference genes. Our research identified the most reliable reference genes for RT-qPCR, establishing a crucial foundation for standardizing gene expression analysis in *D. moldavica*.

## 2. Materials and Methods

### 2.1. Plant Materials and Treatments

The seeds of *D. moldavica* were collected from Luochuan County, Shaanxi Province, China. They were cultivated under controlled conditions at a temperature of 25 °C, with a photoperiod of 18 h light and 6 h dark in a psychrometric room for more than four months until they reached full plant maturity. Then, the roots, stems, leaves, and sepals were collected at the commencement of the flowering stage. The flowers were obtained from the bud, initial flowering, and full flowering stages, respectively. The samples were randomly collected from five plants. Three independent biological replicates were performed.

The same seeds as previously mentioned were sown and allowed to grow for eight weeks until the leaves had fully expanded. The plants were then subjected to varying concentrations of PEG6000 treatment to induce drought conditions, based on our previous research. In the preliminary experiment, we set five concentration gradients of PEG (0% (control, treated with sterile distilled water), 5%, 10%, 15%, and 20%) and four durations (0 h, 24 h, 48 h, and 72 h) for sampling. After 48 h, the plants treated with 15% PEG6000 appeared to wilt, and the fresh weights of the whole seedings were significantly different than the other concentrations. Subsequently, the roots and leaves were harvested for further analysis. Each treatment was biologically replicated three times. The collected root and leaf samples were rapidly frozen in liquid nitrogen and stored at −80 °C for subsequent experiments.

### 2.2. RNA Isolation and cDNA Preparation

First, 0.1 g of total RNA was isolated from the aforementioned samples using the RNAprep Pure Plant Kit (Tiangen, Beijing, China), following the manufacturer’s instructions. The concentration of the isolated RNA was detected using a TECAN Infinite 200 PRO (TECAN, Männedorf, Switzerland). The integrity of RNA bands was observed via 1% (w/v) agarose gel electrophoresis. Then, 200 ng RNA was used for cDNA synthesis according to the manufacturer’s protocol of the PrimeScript (TM) RT Reagent Kit with a gDNA Eraser (TaKaRa, Tokyo, Japan).

### 2.3. Reference Genes Selected and Primer Design

We identified potential candidate genes from the transcriptome dataset of *D. moldavica* roots and leaves treated with the PEG6000 based on gene annotations. Then, we utilized the FPKM values to identify effectively expressed genes and further verified them using BLAST at NCBI (https://blast.ncbi.nlm.nih.gov/Blast.cgi, accessed on 21 May 2024). Finally, a total of 12 candidate sequences were obtained as potential reference genes for further analysis (Table S1). According to BLAST analysis, it was determined that these gene sequences exhibited the highest similarity with *Salvia miltiorrhiza* and *Salvia hispanica*. Then, the CDS sequences were employed to design gene primers using Primer Premier 6.0 software. Each primer was designed according to the following principle: the length of PCR amplified gene fragment ranged from 100 to 250 bp, the annealing temperature was approximately 60 °C, and the GC content fell within the range of 30–40%. These primers were synthesized by Sangon Biotech (Shanghai, China). More details regarding these candidate reference genes and primers are shown in Table 1 and Table S2.

### 2.4. RT-qPCR Analysis

Firstly, PCR amplification was utilized to validate the accuracy and specificity of the primers for candidate reference genes. Following Liu’s protocol [27], a 20 µL reaction mixture was prepared consisting of 1 µL each of 10 µM forward and reverse gene-specific primers, 1 µL of cDNA templates, 7 µL of 2 × Rapid Taq Master Mix (TaKaRa, Tokyo, Japan), and 10 µL of ddH_2_O. The amplification program consisted of pre-denaturation at 95 °C for 3 min followed by 30 cycles: 95 °C for 30 s, 60 °C for 30 s, and 72 °C for 30 s, with a final elongation at 72 °C for 60 s. The PCR products were determined by 1% (w/v) agarose gel electrophoresis.

Subsequently, RT-qPCR was performed using a LightCycler 96 Real-time PCR Instrument (Roche, Basel, Switzerland) with TB Green^®^ Premix Ex Taq^TM^ II (Tli RNaseH Plus) (TaKaRa, Japan). The total reaction volume was 20 µL, comprising 10 µL TB Green Premix Ex Taq II, 1 µL each of 10 µM forward and reverse gene-specific primers, 2 µL template (first-strand cDNA), and 6 µL ddH_2_O. Amplifications were carried out with initial denaturation at 95 °C for 30 s, followed by 40 cycles of RT-qPCR with three-segment amplification, including 5 s of denaturation at 95 °C, 30 s of annealing at 60 °C, and 10 s of extension at 72 °C for polymerase elongation. Then, the melting step was performed with slow heating, starting at 65 °C with a rate of 0.2 °C per second up to 95 °C, with continuous measurement of fluorescence. The RT-qPCR analysis was conducted in triplicate for three biological replicates.

### 2.5. Gene Expression Stability Analysis

The expression stabilities of the candidate reference genes in the total samples were evaluated using four software programs: Delta Ct (version 1.0), geNorm (version 3.5) [34], NormFinder (version 0.953) [35], and BestKeeper (version 1.0) [36].

The geNorm software is designed for screening and determining the optimal number of reference genes. The stability of each candidate reference gene is represented by the M value, with smaller values indicating higher stability. The pairwise variations are calculated to determine the Vn/n + 1, with a threshold of 0.15. A pairwise ratio (Vn/n + 1) less than 0.15 indicates the required number of genes (n), and another reference gene for correction is not needed. Therefore, the geNorm software allows for the selection of two or more reference gene combinations to correct the data [34]. In contrast, the NormFinder software identifies only one optimal reference gene [35]. The BestKeeper software compares the expression levels of up to ten reference genes and ten target genes in a maximum of 100 samples, using the coefficient of variation (CV) and standard deviation (SD) to assess reference gene stability [36].

Finally, the RefFinder program (http://blooge.cn/RefFinder/, accessed on 22 April 2022) was used to comprehensively verify the expression stability of the 12 candidate reference genes [37]. The Venn diagrams were generated using TBtools-II (version 2.026) [38].

### 2.6. Validation of Candidate Reference Genes

The PEG6000 treatment was used to simulate the drought experiment to respond to drought. Dehydration-responsive element binding proteins (DREB) are essential in the transcriptional activation of the abscisic acid (ABA)-independent drought stress response [39]. Nine-cis-epoxy carotenoid dioxygenase (NCED) is a key enzyme required for abscisic acid (ABA) biosynthesis in the regulation of ABA-dependent drought stress responses [40]. The two genes *DREB* and *NCED* were used as the target genes to verify the stability of the selected reference genes under 15% PEG6000 treatment. The primer sequences of the two genes are listed in Table S3. The relative expression levels of genes were calculated using the 2^−ΔΔCt^ method [41], and RT-qPCR analysis was conducted with three biological replicates. Statistical analysis was performed using the SPSS v29.0 software package (SPSS Inc., Chicago, IL, USA). Significant differences between controls and treated samples were calculated at *p* < 0.01 using Student’s *t*-test.

## 3. Results

### 3.1. Expression Profiles of 12 Candidate Reference Genes

The PCR and RT-qPCR were utilized to assess the specificity of the 12 candidate reference genes’ primers. Gel electrophoresis revealed a single band, indicating specific amplification of the primers with the desired size (Figure S1). Additionally, the melting curves generated by RT-qPCR exhibited single peaks, suggesting the primers’ specificity (Figure S2).

**Figure 1:**
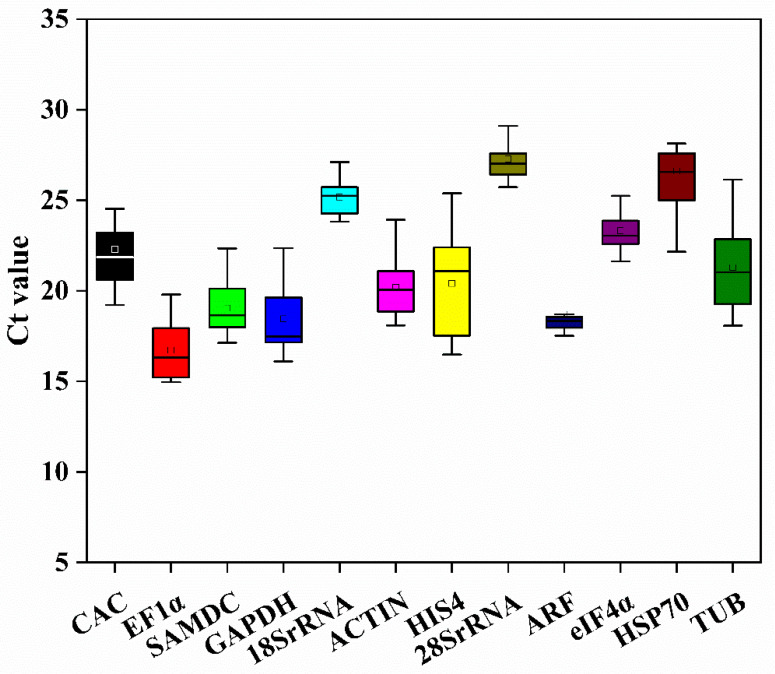
The mean Ct values of the 12 candidate reference genes in *D. moldavica*. The boxes indicate the interquartile ranges, with the median Ct values indicated by the lines in the centers of the boxes. The upper and lower horizontal lines indicate the maximum and minimum values, respectively, and the small squares represent the average values.

To provide an initial assessment, the average cycle threshold (Ct) values of the 12 candidate reference genes in *D. moldavica* were analyzed using all test samples. As shown by the results in Figure 1, the average Ct values ranged from 16.73 to 27.28, indicating differential expression levels of different treatments and different tissues in *D. moldavica*. Among all samples, *EF1α* showed the lowest average Ct value (16.73), followed by *GAPDH* (18.45) and *ARF* (18.72), while *28S rRNA* displayed the highest Ct value (27.28). The median Ct values for *EF1α*, *GAPDH*, and *ARF* were 16.31, 17.49 and 18.34, respectively. Furthermore, *18S rRNA* (23.82–27.11), *eIF4α* (21.63–25.25), and *ARF* (17.51–21.53) showed a narrower range of Ct values, indicating a more stable expression level. *HIS4* (25.38–16.48) had the highest variation, representing that its stability was the worst. However, further research will be required in order to thoroughly analyze the stability of these genes.

### 3.2. Expression Stability of 12 Candidate Reference Genes

#### 3.2.1. Delta Ct Analysis

**Figure 2:**
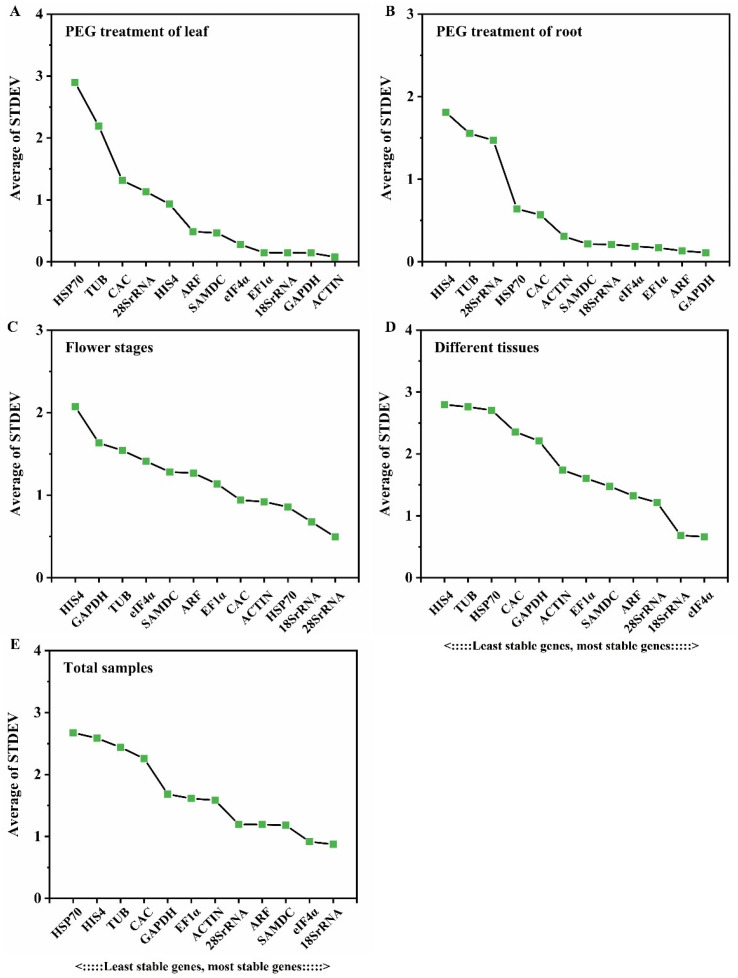
The average standard deviation (STDEV) was calculated according to the delta Ct of 12 candidate reference genes in *D. moldavica*. Results from (**A**): after the PEG6000 treatment the leaves were collected for the extraction of RNA; (**B**) after the PEG6000 treatment, the roots were collected for the extraction of RNA; (**C**) at three flower development stages (the bud, initial flowering, and full flowering stage); (**D**) in different tissues, including roots, stems, leaves, flowers, seeds, and sepals; (**E**) the total samples.

The delta Ct (ΔCt) method was utilized to compare the relative expression levels of “gene pairs” in the samples of each group. The stabilities of the candidate reference genes were ranked based on the repeatability of the mean standard deviations of gene expression differences (STDEV) between samples [42,43]. The results demonstrated that the mean STDEV values of different candidate reference genes varied across different experimental conditions (Figure 2). Specifically, under PEG treatment, the reference gene *ACTIN* (0.08) showed the lowest average STDEV value, suggesting the highest stability (Figure 2A). Similarly, in the case of roots collected under PEG treatment, *GAPDH* (0.11) demonstrated the lowest average STDEV value, indicating the highest stability (Figure 2B). In the context of different flower development stages, *28S rRNA* (0.49) displayed the smallest STDEV value (Figure 2C). Furthermore, in different tissues, *eIF4α*, with the smallest average STDEV value of 0.66, was identified as the most stable gene (Figure 2D). In the overall sample set, *18S rRNA* (0.87) exhibited the smallest STDEV value (Figure 2E). Conversely, *HIS4* and *HSP70* showed the highest average STDEV values among the determined samples.

#### 3.2.2. GeNorm Analysis

**Figure 3:**
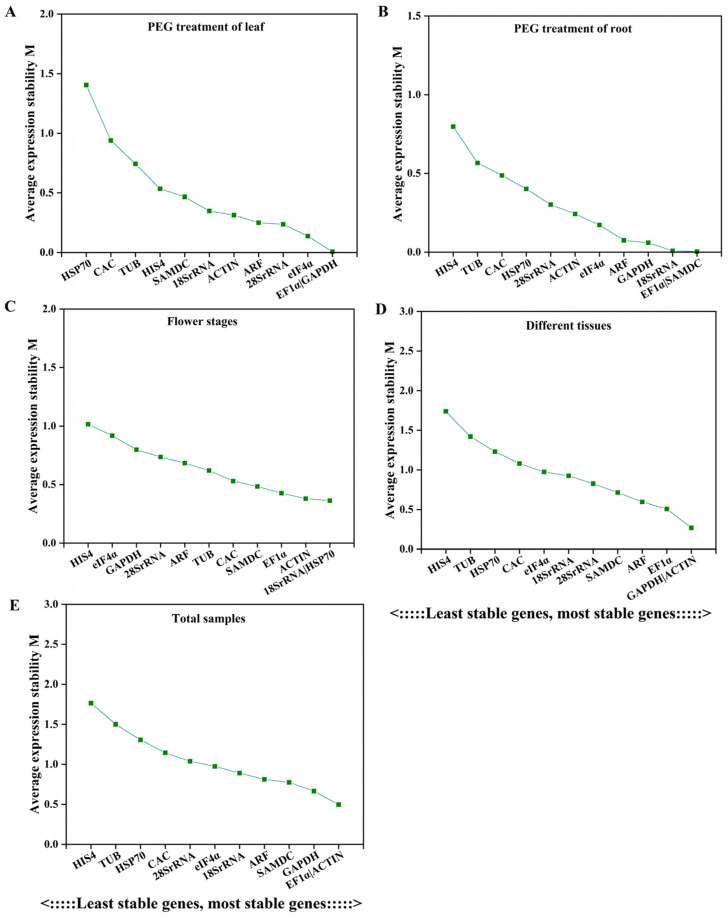
Average expression stability and M value analysis by geNorm. Results from (**A**): after the PEG6000 treatment, the leaves were collected for the extraction of RNA; (**B**) after the PEG6000 treatment, the roots were collected for the extraction of RNA; (**C**) at three flower development stages (the bud, initial flowering, and full flowering stage); (**D**) in different tissues, including roots, stems, leaves, flowers, seeds, and sepals; (**E**) the total samples.

The mean stabilities determined according to the expressions of the 12 reference genes were represented by determining the M values using geNorm (Figure 3). An M value below the threshold of 1.5 was considered stable, with a lower M value indicating greater stability of the reference gene [34]. Significant differences in M values were observed among the 12 reference genes in various samples, indicating variations in their expression stabilities (Figure 3). Specifically, *EF1α* and *GAPDH* were found to be the most stably expressed genes in leaves under PEG6000 treatment (Figure 3A), while *EF1α* and *SAMDC* exhibited the highest stability in root samples under the same treatment (Figure 3B). Additionally, *18S rRNA* and *HSP70* were identified as the most stable genes across different stages of flower development (Figure 3C), and *GAPDH* and *ACTIN* showed the most stable expression levels in different tissues (Figure 3D). Overall, *EF1α* and *ACTIN* demonstrated the most stable expression levels across all samples (Figure 3E). In most cases, *HIS4* was consistently identified as the least stable reference gene, which is consistent with the results of the ∆Ct analysis.

**Figure 4:**
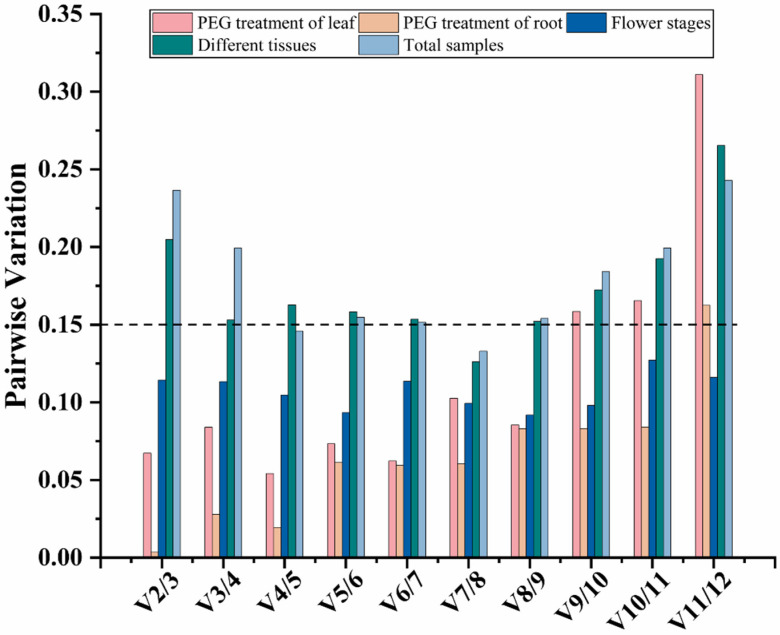
Pairwise variation values calculated by geNorm from the 12 candidates in *D. moldavica*. The Vn/n + 1 function of geNorm was employed with a threshold of 0.15 to determine the optimal genes.

Using multiple reference genes as a standardized internal control is recommended in order to improve the accuracy of RT-qPCR data [10,21,34]. The Vn/n + 1 function of geNorm was employed with a threshold of 0.15 to determine the optimal genes (Figure 4). In leaf samples under the PEG6000 treatment, the ratios from V2/3 to V8/9 were all less than 0.15, suggesting that two suitable reference genes were adequate for standardizing the treatment data. Similarly, in root samples under the PEG6000 treatment, the ratios from V2/3 to V10/11 ratios were less than 0.15, suggesting that two suitable reference genes were sufficient to standardize the treatment data. For the samples of flower stages, the ratios were all less than 0.15, indicating that two suitable reference genes were suitable for standard gene expression. For different tissues and total samples, the V7/8 values were less than 0.15, indicating that seven was the optimal number of reference genes for each sample type.

#### 3.2.3. NormFinder Analysis

The stabilities of the expressions of 12 candidate reference genes were assessed using NormFinder, with lower stability values indicating greater stability. The stability values for the 12 candidate reference genes are presented in Table 2. According to the NormFinder evaluation, *SAMDC* (0.018) and *GAPDH* (0.005) were identified as the most stable genes in leaves and roots under the PEG6000 treatment, respectively. *HSP70* (0.092) ranked highest across three flower development stages. Interestingly, *ARF* (0.246 and 0.256) exhibited the highest stability in different tissues and in the total samples. In most cases, *HIS4* (1.348, 0.954, 2.196, and 1.957) was found to be the least stable gene in the tested samples, which is consistent with the results of the ∆Ct and geNorm analyses.

#### 3.2.4. BestKeeper Analysis

The BestKeeper software was utilized to analyze the expression of the 12 candidate reference genes through the calculation of the standard deviation (SD) and the coefficient of variance (CV) of the average Ct values in the RT-qPCR analysis (Pfaffl et al. 2004) [36]. The most stable genes exhibited the lowest SD ± CV values, with SD values below 1. In Table 3, *ACTIN* (0.06 ± 0.34) showed the highest stability in PEG-treated leaf samples, while *EF1α* (0.06 ± 0.41) showed the highest stability in PEG-treated root samples. Additionally, *28S rRNA* (0.38 ± 1.42) displayed the most stable expression across three flower development stages, and *18S rRNA* (0.59 ± 2.35; 0.76 ± 3.01) showed the most stable expression in different tissues and in the overall samples.

### 3.3. Comprehensive Stability Analysis of the Reference Genes by RefFinder

**Figure 5:**
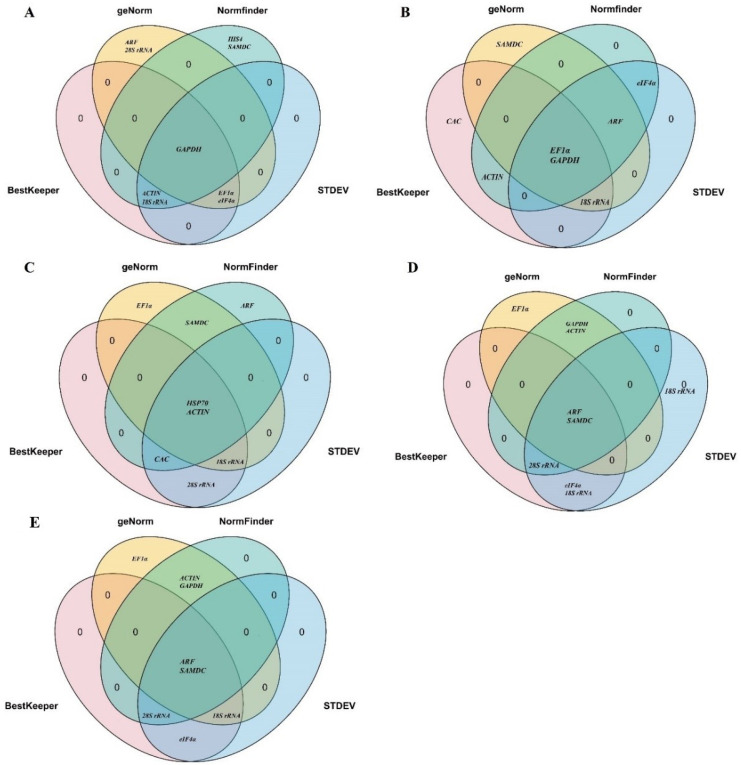
A comprehensive analysis of the top five stable reference genes ranked by four programs. (**A**) The leaf samples under PEG6000 treatment; (**B**) the root samples under PEG6000 treatment; (**C**) at three flower development stages (bud, initial flowering, and full flowering stages); (**D**) in different tissues, including roots, stems, leaves, flowers, seeds, and sepals; (**E**) the overall samples. The intersection of the sets from the four programs indicated that the genes were consistently identified as the most stable across all programs.

This study reveals that the algorithms of each software utilized varied, leading to discrepancies in the assessment of reference gene stability. To mitigate the disparities, a comprehensive analysis of four programs (∆Ct, geNorm, NormFinder, and BestKeeper) was conducted to identify the most suitable reference genes. The comprehensive ranking outcomes for the five most stable reference genes are shown in Figure 5 and Table S3. In the context of leaf samples subjected to PEG6000 treatment, *GAPDH* emerged as the most stable reference gene (Figure 5A). Conversely, in root samples under the same treatment, *EF1α* and *GAPDH* were identified as the most stable reference genes (Figure 5B). During different stages of flower development, *HSP70* and *ACTIN* were deemed the most stable reference genes (Figure 5C). Furthermore, in diverse tissues and overall samples, *ARF* and *SAMDC* exhibited the highest stability (Figure 5D,E).

RefFinder was typically used to comprehensively evaluate the stability of the 12 reference genes, following delta Ct, geNorm, NormFinder, and BestKeeper, to determine the optimal reference gene [44,45]. Upon combining the RefFinder analysis to validate the above comprehensive ranking results, the results were obtained, and are shown in Table 4. In leaf samples under the PEG6000 treatment, *ACTIN* was confirmed as the most stable reference gene, followed by *EF1α*. In root samples under the PEG6000 treatment, *EF1α* was the most stable reference gene, followed by *eIF4α*. Notably, *HSP70* was established as the most stable reference gene during different stages of flower development. In different tissues and the overall samples, *ARF* was the most stable reference gene. Through these two comprehensive analysis methods, the results were largely consistent, except for the different outcomes in leaf samples under PEG6000 treatment (Figure 5, Table 4 and Table S4). The geNorm software suggested that in leaf and root samples under PEG6000 treatment, the optimal number of reference genes was two. Therefore, in leaf samples under PEG6000 treatment, the stable reference genes were *ACTIN* and *EF1α*; in root samples under PEG6000 treatment, the stable reference genes were *EF1α* and *GAPDH*.

### 3.4. Validation of the Stability of Reference Genes

**Figure 6:**
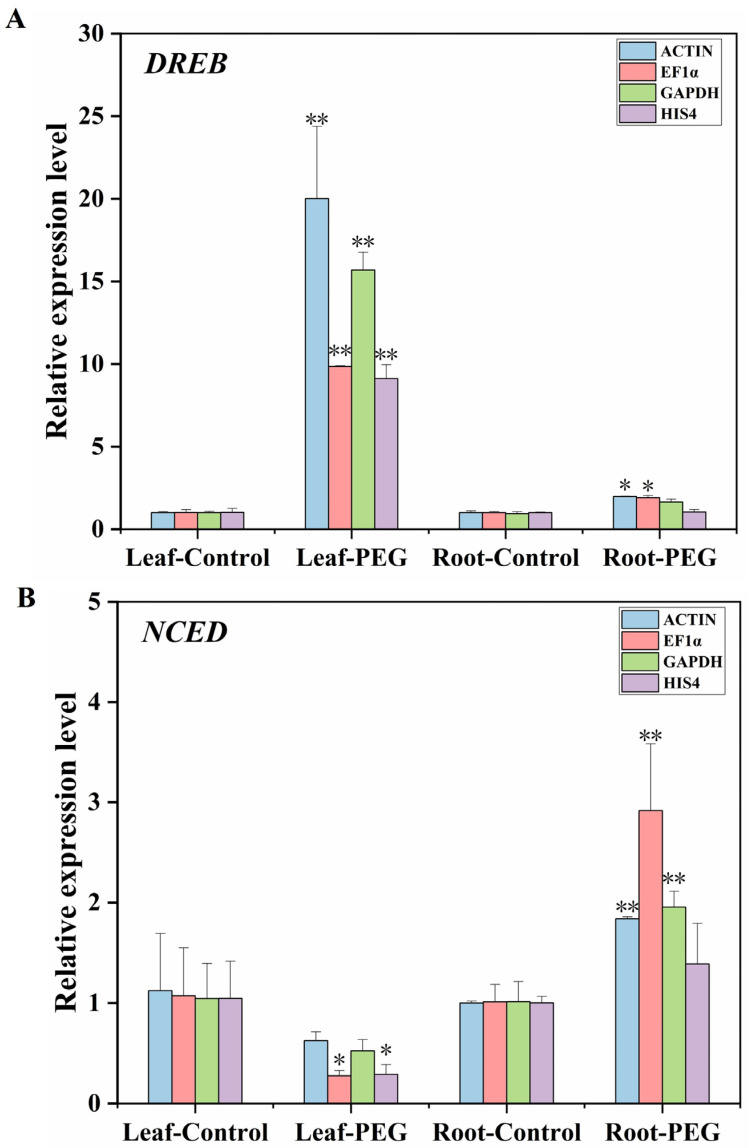
Relative expression levels of *DREB* and *NCED* under PEG6000 treatment in leaves and roots. The results were normalized with three stable genes (*ACTIN*, *GAPDH*, and *EF1α*) and one unstable reference gene (*HIS4*). (**A**) The relative expression levels of *DREB*; (**B**) the relative expression levels of *NCED*. The samples treated at 0 concentration for 0 h were used as controls. Error bars indicate the standard deviations (±SD) from three biological replicates. Significance differences analysis was performed using the *t*-test (* *p* < 0.05, ** *p* < 0.01).

According to the above four methods and comprehensive analysis, we determined that *EF1α* and *GAPDH* were stable reference genes in leaves and roots under PEG6000 treatment (Figure 5), and *ACTIN* and *EF1α* were stable reference genes in leaves and roots under PEG6000 treatment (Table 4). Therefore, *ACTIN*, *EF1α*, and *GAPDH* were selected to verify the expression of the two drought stress response genes, *DREB* and *NCED*. In contrast, the less stable gene *HIS4* was utilized for assessing the credibility of the reference genes in *D. moldavica*. In Figure 6A, it is shown that the expression trend of the DREB gene was extremely significantly increased in the PEG6000-treated leaves. The expression trend of the DREB gene was also increased when using *ACTIN*, *EF1α*, and *GAPDH* as internal reference genes in the samples of PEG6000-treated roots, while the *HIS4* as an internal reference gene the expression trend of the DREB gene was basically unchanged. Similarly, the expression trend of the *NCED* gene was extremely significantly increased in the PEG6000-treated roots when using *ACTIN*, *EF1α*, and *GAPDH* as reference genes, but the trend of the change was not significant when using *HIS4* (Figure 6B). Therefore, the selection of reference genes had a direct influence on the expression of the target genes.

## 4. Discussion

In the field of gene expression research, RT-qPCR is frequently employed due to its high sensitivity, strong repeatability, exceptional specificity, and ability to conduct high-throughput analysis. This approach involves utilizing amplification data from dependable reference genes and target genes to ascertain the expression levels of the latter [10,46]. *D. moldavica* possesses significant medicinal and ornamental properties, yet its molecular biology has been relatively underexplored. Currently, there is a lack of comprehensive research on reference genes, which hinders the advancement of gene function and genetic studies. Consequently, it is imperative to undertake the selection and identification of reliable reference genes for RT-qPCR in *D. moldavica*. Ideally, the stability of the reference gene should not be influenced by the experimental conditions. Studies on screening stable reference genes are generally affected by genotype, various tissues, growth and development, and biotic and abiotic treatments [28,47]. In this study, a total of 12 candidate reference genes from our transcriptome database were utilized to evaluate and confirm their stability for further gene expression research in *D. moldavica*.

Up to now, the genome of *D. moldavica* has not been publicly disclosed. We identified 12 candidate reference genes by analyzing the annotations and FPKM values from a full-length and transcriptome dataset generated from various tissues and simulated drought treatment samples to assess the levels of gene expression. Liu et al. obtained the candidate reference genes based on FPKM values greater than 50 [27]; Zhang et al. did the same according to the variable coefficient of FPKM [44]. To screen more reference genes, we selected the FPKM with a numerical value and stable expression levels in all test samples. To verify the accuracy and specificity of the primers of the 12 candidate reference genes, the products amplified by PCR were detected as a single band via agarose gel electrophoresis (Figure S1). The dissolution curve of 12 candidate reference genes showed single narrow peaks, indicating the primers had high specificity (Figure S2). This yielded preliminarily evaluated Ct values of the total samples: the average Ct values of the 12 reference genes ranged from 16.73 (*EF1α*) to 27.28 (*28S rRNA*) (Figure 1). In *Cryptomeria fortune*, the average Ct values varied from 6.241 to 26.958 [19], while in *Stipagrostis pennata*, the average Ct values varied from 16.89 to 29.12 [10]. This indicated that the Ct values in the test samples were diverse, likely influenced by biotic and abiotic stresses, as well as the different organs and development stages. Therefore, it is necessary to select appropriate reference genes for gene standardization under specific experimental conditions.

There were four different statistical programs (∆Ct, geNorm, NormFinder, and BestKeeper) used to analysis the stabilities of the candidate reference genes. The screening of unstable reference genes was consistent across the four algorithms for the 12 candidate reference genes. However, discrepancies were observed in the evaluation of the most stable reference genes, as each software operated based on its principles. For example, in the case of leaves treated with PEG6000, *ACTIN* was the most stable reference gene according to ∆Ct and BestKeeper, while geNorm indicated *EF1α* and *GAPDH* to be the most stable. NormFinder, on the other hand, identified *SAMDC* as the most stable reference gene. Similarly, in the case of roots treated with PEG6000, *GAPDH* was identified as the most stable reference gene by ∆Ct and NormFinder, while geNorm and BestKeeper indicated *EF1α* to be the most stable gene. A comprehensive analysis using a Venn diagram for the top five stable reference genes selected by four algorithms revealed that *GAPDH* was the most stable reference gene under PEG6000 treatment of leaves, while *EF1α* and *GAPDH* were the most stable reference genes under PEG6000 treatment of roots (Figure 5, Table 4 and Table S4). In *Stipagrostis pennata*, *GAPDH* was also the most stable reference gene under drought stress [10], while under salt and drought stress, *EF1α* and *TUB3* were the most stable in the halophyte *Halostachys capsica* [45].

In the examination of samples characterized by three stages of flower development to assess the stability of reference gene expression, four different programs were utilized. The results indicated that *28S rRNA* was the most stable gene according to ∆Ct and BestKeeper analyses, while *HSP70* was deemed the most stable gene by NormFinder. Additionally, geNorm analysis identified *18S rRNA* as the most stable gene. With comprehensive analysis using a Venn diagram and RefFinder, the result was that *HSP70* was the most stable gene. In barley, *HSP70* was the most stable gene throughout micro-malting in different varieties [48], and for low-temperature-stress leaves, *HSP70* was suitable as the reference gene [29].

In the assessment of reference gene expression stability in different tissues, *eIF4α* was found to be the most stable reference gene according to ∆Ct analysis, while geNorm analysis identified *GAPDH* as the most stable reference gene. NormFinder and BestKeeper analysis revealed that *ARF* and *18S rRNA* were the most stable reference genes, respectively. With comprehensive analysis by Venn diagram and RefFinder, the result showed that *ARF* was the most stable gene (Figure 5, Table 4 and Table S4). Previous studies have also demonstrated that *ARF* is a candidate reference gene in sweet potato in different tissues, i.e., leaves and roots, as well as under four different environmental stress treatments, i.e., cold, drought, salt, and oxidative stress [31]. Similarly, other studies have reported that *EF1α* is a stable reference gene for different developmental stages of *Bambusa tulda* [49].

## 5. Conclusions

After conducting a comprehensive analysis, it was determined that *ACTIN* and *EF1α* exhibited the highest stability as reference genes during the treatment of leaves with PEG6000. Similarly, *EF1α* and *GAPDH* were identified as the most stable reference genes during the treatment of roots with PEG6000. Furthermore, *HSP70* was the most stable gene across various stages of flower development, while *ARF* demonstrated the highest stability in different tissues and in the total samples. It was observed that the suitability of each reference gene varied under different experimental conditions. Notably, *ACTIN*, *GAPDH*, *EF1α*, *HSP70*, and *ARF* were identified as suitable reference genes for investigating gene expression in *D. moldavica*. The stability of these reference genes, as established in this study, serves as a valuable foundation for future research on gene expression and molecular biology in *D. moldavica*.

## Figures and Tables

**Table 1:** The information of 12 candidate reference genes obtained from RNA-Seq libraries of *D. moldavica*.

Gene Name	Gene ID	Expression (FPKM)				Gene Annotation
		Leaf		Root		
		Control	PEG	Control	PEG	
*18S rRNA*	c100609	10.37	9.46	10.77	8.07	18S ribosomal RNA
*ACTIN*	c110032	669.04	393.55	519.34	512.14	Actin
*HIS4*	c103664	118.31	172.25	181.46	153.77	Histone 4
*HSP70*	c112390	267.91	77.51	44.82	39.42	Heat shock protein 70
*28S rRNA*	c117403	4.97	3.89	5.58	4.14	28S ribosomal RNA
*ARF*	c111206	613.04	488.67	608.69	599.65	ADP-ribosylation factor
*CAC*	c100772	37.13	73.32	61.93	64.44	Clathrin adaptor complex
*EF1α*	c118069	2997.05	1986.06	2565.10	2184.92	Elongation factor 1-alpha
*SAMDC*	c102943	457.44	236.29	374.79	324.16	S-adenosylmethionine decarboxylase
*GAPDH*	c118460	449.81	274.44	485.34	452.65	Glyceraldehyde 3-phosphate dehydrogenase
*eIF4α*	c118665	21.25	7.11	15.18	13.63	ATP-dependent RNA helicase eukaryotic initiation factor 4-α
*TUB*	c117718	85.25	133.54	55	63.32	tubulin alpha-3

**Table 2:** Expression stability values in *D. moldavica*, determined with NormFinder.

Ranking	PEG6000 Treatment of Leaf	PEG6000 Treatment of Root	Flower Stages	Different Tissues	Total Samples
1	*SAMDC* 0.018	*GAPDH* 0.005	*HSP70* 0.092	*ARF* 0.246	*ARF* 0.256
2	*HIS4* 0.018	*ARF* 0.005	*CAC* 0.139	*28S rRNA* 0.263	*SAMDC* 0.397
3	*18S rRNA* 0.024	*eIF4α* 0.038	*SAMDC* 0.273	*ACTIN* 0.399	*ACTIN* 0.478
4	*ACTIN* 0.024	*ACTIN* 0.043	*ARF* 0.373	*SAMDC* 0.558	*GAPDH* 0.505
5	*GAPDH* 0.126	*EF1α* 0.163	*ACTIN* 0.388	*GAPDH* 0.571	*28S rRNA* 0.628
6	*EF1α* 0.138	*SAMDC* 0.167	*TUB* 0.404	*EF1α* 0.633	*EF1α* 0.640
7	*eIF4α* 0.374	*18S rRNA* 0.176	*18S rRNA* 0.433	*18S rRNA* 0.830	*18S rRNA* 0.695
8	*28S rRNA* 0.550	*28S rRNA* 0.208	*EF1α* 0.453	*CAC* 0.840	*eIF4α* 0.738
9	*ARF* 0.571	*HSP70* 0.478	*28S rRNA* 0.591	*eIF4α* 0.869	*CAC* 0.931
10	*TUB* 0.926	*CAC* 0.607	*GAPDH* 0.770	*HSP70* 1.136	*HSP70* 1.294
11	*CAC* 1.465	*TUB* 0.746	*eIF4α* 0.905	*TUB* 1.297	*TUB* 1.371
12	*HSP70* 2.588	*HIS4* 1.348	*HIS4* 0.954	*HIS4* 2.196	*HIS4* 1.957

**Table 3:** Expression stability values of candidate reference genes, calculated by BestKeeper.

Ranking	Samples														
	PEG Treatment of Leaf			PEG Treatment of Root			Different Flower Stages			Different Tissues			Total Samples		
	Gene Name	SD	CV [%]	Gene Name	SD	CV [%]	Gene Name	SD	CV [%]	Gene Name	SD	CV [%]	Gene Name	SD	CV [%]
1	*ACTIN*	0.06	0.34	*EF1α*	0.06	0.41	*28S rRNA*	0.38	1.42	*18S rRNA*	0.59	2.35	*18S rRNA*	0.76	3.01
2	*GAPDH*	0.11	0.61	*ACTIN*	0.14	0.76	*18S rRNA*	0.61	2.33	*eIF4α*	0.60	2.58	*eIF4α*	0.77	3.32
3	*18S rRNA*	0.11	0.47	*18S rRNA*	0.14	0.59	*HSP70*	0.73	2.69	*ARF*	0.93	4.96	*ARF*	0.87	4.64
4	*EF1α*	0.12	0.78	*CAC*	0.15	0.78	*ACTIN*	0.77	3.62	*28S rRNA*	1.02	3.71	*28S rRNA*	0.93	3.41
5	*eIF4α*	0.25	1.09	*GAPDH*	0.18	1.04	*CAC*	0.81	3.46	*SAMDC*	1.27	6.61	*SAMDC*	1.09	5.71
6	*ARF*	0.41	2.25	*ARF*	0.24	1.31	*EF1α*	0.99	1.03	*ACTIN*	1.27	6.29	*ACTIN*	1.26	6.25
7	*SAMDC*	0.43	2.33	*eIF4α*	0.27	1.17	*SAMDC*	1.03	5.37	*EF1α*	1.31	7.84	*EF1α*	1.37	8.18
8	*28S rRNA*	0.93	3.31	*SAMDC*	0.30	1.61	*ARF*	1.11	5.83	*CAC*	1.70	7.51	*GAPDH*	1.52	8.23
9	*CAC*	1.20	5.82	*HSP70*	0.78	3.40	*eIF4α*	1.14	4.81	*GAPDH*	1.77	9.39	*CAC*	1.64	7.38
10	*HIS4*	1.79	7.45	*28S rRNA*	1.40	5.11	*TUB*	1.32	6.59	*HSP70*	1.84	6.67	*HSP70*	1.91	7.16
11	*TUB*	1.82	8.64	*TUB*	2.38	10.84	*GAPDH*	1.43	7.74	*TUB*	2.30	10.58	*TUB*	2.13	10.01
12	*HSP70*	2.63	10.58	*HIS4*	2.47	10.54	*HIS4*	1.82	9.72	*HIS4*	2.30	11.17	*HIS4*	2.45	11.87

**Table 4:** A comprehensive ranking of the 12 candidate reference genes, calculated by RefFinder.

Ranking	PEG Treatment of Leaf	PEG Treatment of Root	Different Flower Stages	Different Tissues	Total Samples
1	*ACTIN*	*EF1α*	*HSP70*	*ARF*	*ARF*
2	*EF1α*	*eIF4α*	*SAMDC*	*EF1α*	*ACTIN*
3	*18S rRNA*	*GAPDH*	*ACTIN*	*ACTIN*	*EF1α*
4	*GAPDH*	*ARF*	*EF1α*	*28S rRNA*	*18S rRNA*
5	*eIF4α*	*ACTIN*	*CAC*	*18S rRNA*	*SAMDC*
6	*SAMDC*	*SAMDC*	*ARF*	*eIF4α*	*eIF4α*
7	*ARF*	*18S rRNA*	*28S rRNA*	*CAC*	*28S rRNA*
8	*HIS4*	*CAC*	*18S rRNA*	*SAMDC*	*GAPDH*
9	*28S rRNA*	*HSP70*	*TUB*	*GAPDH*	*CAC*
10	*CAC*	*28S rRNA*	*eIF4α*	*HSP70*	*HSP70*
11	*TUB*	*HIS4*	*GAPDH*	*TUB*	*TUB*
12	*HSP70*	*TUB*	*HIS4*	*HIS4*	*HIS4*

## Data Availability

Data is contained within the article and Supplementary Materials.

## Supplementary Materials

The following supporting information can be downloaded at: https://www.mdpi.com/article/10.3390/cimb46060375/s1.
