# Supplementary material for: Selection of Reference Genes for Expression Normalization by RT-qPCR in Dracocephalum moldavica L
Source: Curr Issues Mol Biol. 2024 Jun 20;46(6):6284–99. doi: 10.3390/cimb46060375 (PMC11202811; doi:10.3390/cimb46060375)
Supplement: Supplementary file 1 [file cimb-46-00375-s001.zip › cimb-3046438-supplementary.pdf]

Selection of Reference Genes for Expression Normalization by RT-qPCR in *Dracocephalum moldavica* L.

Table S1 The gene sequences of 12 candidate reference genes of *D. moldavica*

**Gene name**	**Gene ID**	**Gene sequence**	***Sequence ID***
18S rRNA	c100609	AAAAAAATTAGCCGCAACCAAAAAGCTCTGTTTTTCTTCAATCAC<br>ACACAAACGAATTGCTAAAGGTGAGCGACGAAAACATAGACATG<br>GGTCACAACAAGCAGCAGAAACAGCGGCGAATCAAGAAAGAAG<br>ACCACCGCCACGGACAGTCTCATCAGCTCGACAGGAAGTGCGAT<br>GGAGAGTCTTTGCCGATTGACCCAGCAGCTGGAGAGGAACAGGA<br>GCACAACGTTCCCGCCGTTCAGCTCGCCATGTGGGATTTTGGGCA<br>GTGTGATGCAAAGAGGTGCACAGGGCGCAAGCTTGCGAGATTTG<br>GATTGTTAAAAGAAATGCGGGTTGGTAGTGGATTTGGAGGAATTG<br>CTTTGAGTCCCATTGGAACACAATGCGTCTCCCAAGCAGATCTTG<br>AATTAATAAAAAGGCGAGGTTTAGCTGTTGTTGATTGCTCATGGG<br>CACGCCTAGATGATGTGCCTTTCTCCAAGCTGCGCTGTGCTGCTC<br>CTCGTCTTTTACCTTGGCTGGTAGCAGCAAATCCCGTAAATTATGG<br>CCGGCCTTGTGAGCTTTCATGCGTTGAGGCGCTAGCTGCAGCTTT<br>GATAATATGTGGAGAAGAAGAAACTGGAAATCTATTACTCAGCAA<br>ATTCAAGTGGGGTCATGCTTTCTTGTCACTGAACGGAGAGCTTCT<br>CAAGGCGTACTCTGAATGCAAGAACGGTGCTGACATGATTGTCGT<br>TCAAAACGAGTGGCTTTCACAGCAAAAGTCACAACCCTCGGATG<br>ACCTCAATAAAGTACAAGGAGAGCCTTCTGGATCTAATAAAGACG<br>AAGATGGATCTTCCAGTGGATCTGAAGACGGGCTCCCTCCGCTTG<br>AAAGGAACATAAATCACTTGTATTTAGAGGAAAGTGGTGAGGAA<br>AGCGAGTGAACTACTTTATACTGCCTCTAAGAGGTGTTATCAACTG<br>GAACAAATATGATCGATAATTTTCGAGGAAAAGAGTTTTTGAGTT<br>TTAACCCCTGAGCTTGCCTCAATGCGTTCTTTCTACTCTTTATGCCT<br>AGTAAATAAGGGCAATCGGACCAGCATGAGAAGTAAGATCTGTTT<br>TCGTGGGACCACCAACCCGAATCTCGTCACTAGGCGTGATCACCC<br>AGAATCCAATGTGAGGATTAGGGCTGATCCATCCGTGCACGTGGT<br>TGTCCTTGTTGTCACACGAGTATTGATACTTGTCGTCCACCTAGAT<br>CATAATAAAAATTCAAACACGGTTATTCTATAGGACTGACCCGAA<br>ACAGAAATGGT	XM_057927769.1
ACTIN	c110032	GTGAATTCCAGCAGCTTCCATTCCGATCATTGATGGCTGGAAGAG<br>GACCTCAGGGCAACGGAATCTCTCGGCACCAATAGTTATGACCTG<br>TCCATCGGGAAGTTCGTAGTTCTTCTCCACAGCAGAGCTGGTCTT<br>TGCCGTCTCGATCTCTTGCTCGTAGTCCAAAGCAATGTAGGCCAG<br>CTTCTCTTTAATGTCTCTCACAATTTCCCGCTCGGCAGTGGTGGTG<br>AATGAGTAGCCTCTTTCTGTCAAGATCTTCATGAGGTGGTCGGTC	XM_057912742.1

		AGATCACGCCCAGCCAGATCGAGACGGAGGATTGCATGAGGGAG<br>TGCATAACCTTCGTAGATTGGGACTGTATGGCTGACACCATCACCA<br>GAGTCGAGAACAATACCTACAAGCAATAGCACAAACCAAACATC<br>AATTCATGATGAAAACTACCTAGAATGGAACTTAGTCTAGATACCT<br>TTTTTTACTGATATGAGTCCAATACATCTAGTTATCTACAGTTTCAA<br>TTCTACACAATCGTGATCGTAATAGGCTTCTCAGTATATGGTGTTTT<br>ACTATCATTAATAAAAATACAATACTGCACTAAGACCGGTTATGCC<br>ACAGCAAGATTACTATGGTTATTCGAACAGAAATAATATCACGAAT<br>CAAGAAAAACGTAAAATAGTTTGGCATTTTCTTACCAGTTGTACG<br>ACCACTGGCGTAAAGGGAGAGAACGGCCTGAATGGCAACATACA<br>TAGCAGGGGCGTTGAATGTCTCGAACATGATCTGAGTCATTTTTTT<br>CACGATTGGCCTTGGGGTTGAGAGGAGCCTCTGTCAAGAGAATC<br>GGGTGCTCTTCTGGGGCCACACGAAGCTCGTTGTAGAATGTGTGA<br>TGCCAAATCTTCTCCATATCATCCCAGTTGCTGACGATTCCGTGCT<br>CAATTGGGTACTTCAACGTCAAAATACCTCTCTTGGATTGAGCTTC<br>GTCACCAACATAGGCATCTTTTTGGCCCATGCCAACCATCACACC<br>AGTGTGACGAGGGCGCCCAACAATGCTAGGAAAGACAGCCCTCG<br>GGGCATCGTCTCCAGCAAATCCAGCCTTGACCATTCCGGTTCCAT<br>TGTCGCAGACGAGGGGCTGAATATCCTCAGCTTCGGCCATTTCTT<br>AATTTGCTCTTTGAAGTTTTGCTGAAAACGGGTGTCGCCCTTTCT<br>CGCTCTAGCTTGAGTTTCTCTCTCCAGTAATAGGAAAAACCTCTGT<br>TTTCCCTTGTCCGAAGTGCCGCCCCTCTCG	
HIS4	c103664	GAACAAATACATCGAAGAATCCAATTTTCACCTCAATTTCTCAAAT<br>TTCAGCTCAAGAGAGAGAAAATGTCTGGAAGAGGAAAGGGAGG<br>CAAGGGATTGGGAAAGGGCGGAGCTAAGCGTCACCGGAAAGTG<br>CTGAGAGACAACATCCAGGGCATCACGAAGCCGGCGATTCGCCG<br>TCTCGCTCGCCGTGGCGGCGTCAAGCGCATCAGCGGCCTCATTTA<br>CGAGGAGACTCGCGGCGTGCTCAAGATCTTTCTCGAGAACGTCAT<br>CCGTGACGCCGTCACCTACACGGAGCACGCTCGCCGGAAAACGG<br>TGACCGCCATGGATGTGGTGTATGCGCTGAAGAGGCAGGGCCGTA<br>CCCTCTACGGTTTCGGCGGCTGAGATCTTAGATTGGTTGTATATGG<br>CCTATCGGTAGAATTAGGGCTTTTTTTTTAGTATTTTGGTATTTTGG<br>ATTTTGGTAGGTTGTGTTTTGTTGTTTACTGGTGCGATCTATCTCAG<br>ATTCAATTGAAATTGGATGATTCATATTTCTCTCGATTTTACTAAAT<br>TTTTGTCTCAGTGTTTTATTTGCCAACCTCTTTAGTTCATCTCCATT<br>GTGATTGAATTAAAGCAATGAAACCGTCTTCGTTTAGTCGAATTGT<br>GAATTCGGTA	XM_057929079.1
HSP70	c112390	TGATGAGTCGAAGCAGGTTTCGTATAAGGTGGTGAAGGATGATAT<br>TGGGAATGTCAAGGTGGAGTGCCCTGCTACTGGGAATTTGTTTGC<br>TCCCGAGGAGATTTCTGCGCAGGTCTTGCGAAAACTTGTAGATGA<br>TGCCTCGAAATTCTTGAACGATAAGGTCACGAAAGCGGTTATTAC<br>AGTGCCAGCCTATTTTAATGATTCACAGAGAACTGCAACGAAAGA<br>TGCTGGTCGTATAGCTGGCTTGGATGTTCTTCGCATTATAAATGAG<br>CCCACAGCTGCTTCTTTGGCTTATGGTTTCGAGAAGAAAAGTAAT	XM_057937588.1

		GAAACCATTCTAGTTTTTGATTTAGGAGGTGGCACATTTGATGTTT<br>CAGTTCTTGAGGTCGGAGATGGAGTTTTTGAGGTGCTTTCCACCT<br>CAGGTGACACACATCTGGGTGGTGATGACTTCGATAAGAGAATTG<br>TCGATTGGCTAGCCGATAGTTTCAAGAGAGACGAAGGCATTGATC<br>TCCTCAAGGACAAGCAGGCGCTCCAACGGCTTACTGAATCTGCTG<br>AGAAGGCAAAGATCGAATTGTCTTCTCTAACTCAAACTAATATAA<br>GTTTGCCATTCATCACTGCTACTGCTGATGGTCCTAAACATATCGA<br>GACAACATTAACGAGAGCTAAATTCGAAGACTTATGCTCCGACTT<br>ACTAGACAGGCTTAAAACTCCTGTCAAAACTGCCTTAGATGATGC<br>TAAGCTTTCGATCAAGGAGATAGACGAAGTGATTCTTGTTGGTGG<br>CTCGACTCGAATCCCTTCTGTCCAAGATCTCGTCAGGAAGATGAT<br>CGGGAAAGATCCTAATGTTACTGTCAATCCAGATGAGGTCGTTGC<br>ACTTGGAGCTGCAGTTCAGGCTGGTGTTTTGGCGGGTGATGTGAG<br>CGATATCGTGCTCTTGGACGTGACTCCTTTATCGCTAGGCTTAGAA<br>ACTTTGGGAGGGGTGATGACGAAGATCATACCAAGAAACACGAC<br>GCTGCCAACCTCAAAGTCGGAGGTCTTCTCCACAGCCGCGGACG<br>GGCAAACAAGCGTGGAGATCAACGTGTTGCAAGGTGAGAGAGA<br>ATTTGTAAGAGACAACAAATCATTAGGAAGCTTCCGTTTGGATGG<br>CATCCCGCCTGCTCCCCGTGGCGTCCCTCAGATAGAGGTGAAGTT<br>CGACATAGACGCCAACGGCATCCTCTCCGTCACAGCTATCGACAA<br>AGGAACAGGAAAGAAGCAAGACATCACGATAACTGGTGCCAGCA<br>CATTGCCTCAAGATGAGGTGGATAGGATGGTAAAAGATGCCGAGA<br>GATACGCGAAGGAAGACAAGGAGAGGAGAGAAGCCATTGACAC<br>GAAGAATCAAGCAGAGTCAGTCGTATATCAGACAGAGAAGCAGT<br>TGAAGGAGCTCGGCGATAAAGCGCCGGCTGCGGTGAAAGAGAA<br>GGTGGAGAGTAAACTGACAGAATTAAAAGATTCTCTCTCTAATGG<br>ATCAACTCAAGCCATCAAGGATGCCATGGCAGCACTCAACCAAG<br>AAGTGATGCAATTAGGGCAATCTTTGTACAGCCAGCCGGGAGCAG<br>CGGCCGATGCTGGAGCTGCCACGTCGGGCGGGTCGAACTCGACT<br>GGTAAATCGGACGGAGACGGAGAAGTCATTGATGCTGATTTCACT<br>GACAGCAGCTGATTATGCTTGTTGAGACAATGTGATGATTTTGTGG<br>GAGATTATGTTAGATAAGCC	
28S rRNA	c117403	GGCTACTTAAAAAAATTGAAACATAAGGGAAAAAAGTATACAGAT<br>CGAATATCAAAATTTTGGGAGATTTTAAAATTTAATGGTTAGTGAG<br>AGCTTTTAAATCGTGTGTAAGGCTGCAATTTAAGGAGTCTGGCCA<br>CGAAAAAGTTGAGAGGTTTTCTACTACGCTTATTCTGAAAATCGT<br>GCTGCTGTGCTTTCCGAACGAACAGAGCAATGAAAAATCCTTCTT<br>TATCTTCTACCAAATCCGTCCGCAACAGATGTTCAGAGCCATCAA<br>ACGTGGGGAGACCGCGACGAGGCCATTGGGGAAAGGATGTCGTG<br>AGTTCGAAGCCATGAGATGATGCGAGATTTAGAACAGAGTTGACA<br>ACATCTTCATTCTCAATTTGATGTATTGAACACGTACTGTAAACGA<br>TTCTCTCCACTGCTGGAAAAGATAGTGCATGTTCAAGGACCTTCT<br>TCTGGAATGCAGCAAGCCCACGAAGCCGCTTTGCGTCAACATGA<br>CCGGACTGGCCTGCACTATGTGATGGAAGTAGATGGTCTAATCTCT	XM_057943758.1

		CGAGAGCAGTCCCAGATCCAGAACATGAAGGATCCAAGAGTATA<br>GCCCGAACCTTTGAATGTGAGGGGTCGGTAGGATCAAGGTTTAAG<br>AAGTCTTCATGCTTAACTTTCACATTCGTTGCGCCGGCCAGTTTGA<br>CATTATTTTTCAAGCGCTCGACTCTTTCCTTGTTAAGTTCGCAGGC<br>TATAATCTTTCCCTTGCCCATCATAAGAGCAGCAAGGTGGACAGTT<br>TTGTTTCCAGGAGCTGCACAAGCATCAATGACCTCCCAATCAGGT<br>TTAGGATCAAGAGCTGCTGCAGCCATGGAGCTTGCTTTACCCTGC<br>AGGAAAACACTTCCACTTATGACTAGAGGATGATTGTGTAAATCA<br>GCACCTGGTGGGAGGATTAGTAAATCAGGGACTGTGTTGTCTTCC<br>TTGACCGCATACTGTTGTCCTAATTCACGTATGACAGATTCAGTGT<br>CGGTCTTTAAAGTATTTACTCGAACGTAACGAGGTTTCCTATCAGA<br>AGTTTTATCAAGAGCCATCAAGTCTTTTACATCGTTCAGTCCCTTG<br>CGCTTTAAAATCCGTGATAGAGCCGATTGCAATGCGTCCTTTTTTA<br>ACATCAGATGCTTTTCAGCATCACCAGTTAATAAATCTTTCTGTCC<br>AAAAAGTAAATCATATGTGATGATATACAATAGCTCGTTCTGCTTC<br>TTCCACTTAGCACTTACAACATCTGCCTCCTCCATAACCTCCTTAA<br>TAACAGAGAGATGCTTGAGAGTTTCGCAAACCAGAGCAAATGTA<br>GCCTTCTTGTTCCTGATGGAAGGGCTGTAAACGAGCGACTTAATC<br>GAGCCGACGGCTTTCCGCCGAGCATCGCCGTGAAGTGCGCTGCG<br>CAACACCTTAGCGGCTTCTCTCCGTGCGAAGTAAGCCGACCTTTC<br>GGCGTTGCTACGCCGCTTCACCTCCGGCTTCTGCTCCTTCCCCGC<br>AGATGAGTTAGCCGTAACCGGCTTCCTCCGCGCCATATCTCGCTGT<br>TTGATTTGTCGGGGTTTAGGGTTTTGATTTTTTCTT	
ARF	c111206	CCTGCATCAAGAAAATTTTCTGCAAACTGCATCAAAAGTTGCCTT<br>ACCAATCAAAAGATGATCAAGCAGTGAAAAAGTTAAAGAGCTAT<br>ACCAAAGGTCGAGAAACGGAATAAGCACGTACTTAGCGGCAGAT<br>TTTCATGAAATTATAATCTTGTAAATGATTCTTTAGTAGATGAAAAA<br>AACTCTTTTTGAGTAGATGTAAACAATAAAAGTATTTCACATAAAA<br>TAGAGATACAGATACTCATTGAATTAAGTACTTTCTAGAGTAAGAT<br>TAGGGAAAATAAAACTTATTAAACACTTATTTCCCATTTAATCCAT<br>GCAAACCGACTATCCTACAAGTGATGTAACGGTGAGGAATCCGCA<br>GCCAGCATCACAGCAGACCAACCTGACCGTTGGTCGTGTAGAAG<br>CAAGATCCAACGGCTGGATTCGCAGGAAACAGCTTCATGACAGT<br>AGGGGCAATCCCGTTGATATAGGCAGTTTTAAGTGGCAGTTTTGA<br>CATTTCAGAAGTAAACGGAGCGCATCCAATATATAATCATTGGTTG<br>ACAATTCTCAATCCTCCTCCCGAAAATTGTACTATTAGTATCTCGC<br>GTTGTTCTCAGGCAGCTCCACATTTCTCTCTCTCGCGCCAGCAGG<br>AAACTTCCTTTCTCTCTCTAGCGTCTCTCCAAATCTACCGTTCTAA<br>GAGATCAAAAGTCAGAAAGGAAATGGGGTTGACATTCACAAAGC<br>TCTTCAGCCGGCTTTTTGCCAAGAAGGAGATGCGTATTTTGATGG<br>TTGGTCTTGATGCTGCTGGTAAGACCACCATCTTGTACAAGCTCA<br>AGCTCGGAGAGATAGTTACTACTATTCCTACCATTGGATTCAATGT<br>GGAGACTGTTGAATACAAGAACATTAGCTTCACTGTTTGGGATGT<br>TGGGGGTCAGGACAAGATCCGCCCATTGTGGAGGCACTACTTCCA	XM_057955006.1

		AAACACTCAGGGTCTCATATTCGTGGTGGATAGCAATGATAGGGA<br>CCGTGTTGTTGAGGCAAGGGATGAATTGCATAGGATGTTGAATGA<br>GGTAATAATCTATTCTGTACTCTGTTCATGTATCAGAACATTATTTAT<br>CATTCAGGACAAGTCATGTAAAGTTTTGGTGCTCTAGGAAATGCT<br>CTCATAAATATTATACTTCATAATTTGTTGGGGCTTTCATGTTATTCA<br>GGGAGTGCCTTTTGAAAGTCTTGCAGCATACACTATTTCTATATATT<br>CTCTACTTATCATAATTAGCAATATAGAGCAATATGCAATAAATTTG<br>AACTTTGTAATGTCTCCTTTACTGATTGATCAGACTGACCGTTTGC<br>TCCATGAGTATATGCACCTCTAGTTTCATTGGTGGTTGACAGGAGT<br>GAGTGATAATATCCATACGATTTTGTCTTTAATCTAGAATGATTTAA<br>TTGTTTGTGCCTCTTTGAGTAGCCTG	
CAC	c100772	GTAGTATACGAAAAAAAGCAGTTCGCTGACTGAAGAATTTTGCAT<br>TTGAATTCAATTCGATCGCAACAAATCTGTTCAACGGAGCAGATC<br>TCGTTTCTCATCTGACTCGATCGGAGCTCCGGCGAGATCTCAGCC<br>ATGCCGGTGGCGGCTTCCGCTATTTACTTCCTTAATCTTCGCGGCG<br>ATGTCCTCATCAATCGCCTCTACCGCGACGACGTCGGTGGCAACA<br>TGGTGGATGCTTTCCGAGTCCATATCATGCAAACAAAGGAACTTG<br>GAACCTGTCCTGTCCGGCAGATTGGGGGATGCTCATTCTTTTACAT<br>GAGAATTAGCAATGTCTATATCGTGATTGTCGTAAGCAGCAATGCA<br>AATGTTGCTTGTGCATTCAAGTTTGTTGTTGAGGCTGTCACACTAT<br>TTAAGTCATATTTTGGTGGATCGTTTGATGAGGATGCCATTCGCAA<br>TAATTTCGTTCTGATATATGAACTGTTGGATGAAATTATGGATTTTG<br>GTTATCCTCAAAATCTTTCTCCTGAAATCTTGAAGCTGTATATAACT<br>CAGGAAGGCGTACGCTCTCCTTTTTCATCCAAGACTGCTGATAAG<br>CCAGTTCCAAATGCAACTTTACAAGTTACTGGTGCTGTTGGGTGG<br>CGCAGAGAGGGCCTTGCATATAAGAAAAATGAGGTATTTCTTGAT<br>ATTGTGGAGAGTGTCAATCTTCTTATGTCTTCAAAAGGTAGTGTTC<br>TTCGCTGTGATGTAACTGGGAAGATTCTTATGAAATGCTTCCTTTC<br>TGGTATGCCTGATTTGAAGTTGGGATTAAATGATAAAATCGGGCTC<br>GAGAAAGAATCACAGCTCAAATCCCGTCCTGCTAAAAGTGGAAA<br>GACTATTGAGCTTGATGATGTCACTTTCCATCAATGTGTGAACCTT<br>ACTAGATTCAATTCGGAAAAGACTGTTAGTTTTGTTCCTCCAGATG<br>GTGAATTTGAATTAATGAAGTATCGTATTACGGAGGGAGTTAATCT<br>TCCATTTCGGGTTCTCCCAACTATCAAGGAACTGGGTCGAACACG<br>TATGGAAGTAAATGTAAAGGTGAAGAGTGTCTTCGGGGCAAAAAT<br>GTTTGCACTTGGCGTGGTTATTAAGATTCCAGTTCCAAAACAAAC<br>AGCAAAAACTAGTTTTCAGGTGACATCAGGCAGGGCAAAGTACA<br>GTCCTTCTATTGACTGTTTGGTTTGGAAGATAAGAAAATTTCCAGG<br>GCAAACTGAGCCAACATTGAGTGCTGAAGTAGAGCTAATATCCAC<br>AATGACTGAAAAGAAGTCTTGGACGCGGCCTCCAATTCAGATGG<br>AATTTCAGGTTCCTATGTTTACAGCATCTGGATTGCGTGTTCGTTT<br>CCTGAAGGTATGGGAAAAAAGTGGCTACAACACCGTTGAATGGG<br>TTCGTTACATTACAAAAGCTGGTTCATATGAAGTTAGATGCTAAGA<br>TAAATACAAGAGTTGTAATGGCATGTTTTGGGAGAAGACAACTAG	MF621044.1

		GCAAAGTACAAGGAAATATTTTCGATATACATGGACCAACGAAGT<br>TTTCTATCTGCAAGATGTATTCTTTAAGATCAAGACTATTCGGTGA<br>AACAAGAAGAAAGACAGGTAATGAGTCCGTGTTGTTCACAGCGG<br>CCGTGTGTACTCGTACAGACTCATTTGTTCATTTGAAGTTTGTTTG<br>CAAAACTTTATTGTATAGTGTAGGCCACATAATTAGGTATGAAATT<br>GTATTATGTATTTTGGAGATTTTGTTAGATGATTGGAACTGTGGAG<br>TTGTCGTTCTATTGATTGTGC	
EF1α	c118069	CCGACAAAAACTTGATATATCATAAAATGATAAAATCAGACAAAA<br>GTCATATACTGACGCCACCGGCGATCCAGAACCCAAGTCTGGGAG<br>CCAAATTCGCACGACAAAGACCAACAAACACGAAATGCAGAAA<br>GAAATAAGTAGTTTTTCTTGCAGAAATAGTAAACAAACAGAAAGA<br>TAATTCTATCATTTCACTTGACCCCCTTCTTGGCTGCTGCCTTGGTC<br>ACCTTGGCACCTGATGGGTCCTTCTTCTCCACACTCTTGATCACAC<br>CAACAGCAACCGTCTGACGCATGTCACGAACAGCAAACCTTCCA<br>AGAGGAGGGTACTGCGAGAAGGTCTCCACCACCATGGGCTTGGT<br>TGGGGTCATCTTCACCATACCAGCATCTCCGTTCTTCAGGAACTTG<br>GGTTCCTTCTCGAGCTCCTTACCCGATCGCCTGTCGATCTTGGTCA<br>AGATCTCAGTGAACTTGACTGCAATGTGGGAGGTGTGGCAATCG<br>AGAACTGGGGCGTATCCGTTTCCAATCTGGCCGGGGTGGTTCATG<br>ATGATGACCTGGGAGGTGAAGTTGGCAGCTTCCTTGGCGGGATCA<br>TCCTTGGAGTTGGATGCAACATAACCACGCTTCAGATCCTTAACA<br>GCAACGTTCTTCACGTTGAACCCCACATTGTCACCAGGAAGAGC<br>CTCCTGCAGGGCCTCGTGGTGCATCTCCACTGACTTAACTTCAGT<br>GGTCAAACCAGATGGGGCAAAAGTGACCACCATTCCAGGCTTAA<br>TCATACCGGTCTCAACACGACCTACAGGCACAGTACCAATACCAC<br>CGATCTTGTAAACATCCTGCAGTGGGAGACGGAGGGGCTTGTCCG<br>ATGGCCTCTTTGGCTCTGAAATGGCATCAAGTGCCTCCAGCAGGG<br>TTGGGCCCTTGTACCAGTCGAGGTTGGTGGACCTCTCAATCATGT<br>TGTCTCCCTCAAATCCAGAAATGGGGACGAATGGGATCTTCTCAG<br>GATTGTATCCAACCTTCTTGAGGTACGAAGCCACTTCCTTAATGAT<br>TTCATCGTACCTAGCCTTGGAGTATTTTGGTGTGGTGGCATCCATC<br>TTGTTGCAGCAGCAAATCATCTGCTTGACTCCAAGAGTGAAAGCA<br>AGCAACGCATGCTCACGGGTCTGACCGTCTTTGGAGATACCAGCC<br>TCAAAACCACCAGTGGTGGAGTCAATGATGAGAACAGCACAGTC<br>AGCCTGTGAGGTACCAGTAATCATGTTCTTGATGAAATCACGATGT<br>CCAGGAGCATCAATGACTGTGCAGTAGTACTTGGTGGTCTCGAAT<br>TTCCACAAGGCAATATCAATGGTGATACCACGCTCGCGCTCAGCC<br>TTCAGTTTGTCGAGCACCCATGCATATTTGAACGACCTCTTGTTCA<br>TCTCAGCAGCCTCCTTCTCGAACCTCTCGATGACACGCTTGTCAA<br>TACCACCAAGCTTGTAAATCAAGTGACCGGTGGTGGTAGACTTTC<br>CAGAGTCGACATGGCCAATGACCACGATACTGATATGAATCTTCTC<br>TTTACCCATATCTCAGCAGTGAAAAGGTATTAATTCTTCAAAAAGT<br>AGATCGAGAGATAAGTAGGGAAGGAGAGACGAAAGGCCT	XM_057926709.1

SAMDC	c102943	GGGCATTGAAGGAAGGAAAATCGAAAGAAGGCCATAACAAACAC<br>ACGATTAATAAAACAAAACTCAATAACAAATTCAGTCTTCAACAT<br>AACATAATTAAAAGAACAGTGCCAACACTTGAAAACAAGAAAGC<br>AATACGATAGTACGAATACTTTATTTAAAAGTAGTAAAGGAAACAC<br>ACAAGCTACTCCTTTTCTTCTTCCTCGTCCTCATCATCGTCTCTGA<br>AACATTTCAGAGTGGACCTCGGAGATCCACAGGTCGCAGCTCCC<br>CTCACAAACTTCTGGTAGGAAATAGCACCACCAGCACCCAGCTCC<br>TCAACGCTCCTCTCTCCACCATCATAACATTTCAAATCCAGCGAGC<br>AGATCTCATCATATAACTCAGCACCACACTGCGCATGCACAGACA<br>CAGAAAACTCATTTGGTTCGAAACAGGTCAAGACCCTCTCGACC<br>AGAGCACGCAGATTCACAGCTTTCAAATCATAACCAGCAGCTTCA<br>AAGCTCGCATAACTGAAACCATCTTCTGGAGTAACATGAATAGTA<br>GACACAGCAGCTCCTTCAATTGCGTTCATGGAGTATCCGCAAGGC<br>TCAAACTCAAAGTCGCAGATGCGAGACTTTGGGAGGATGTTGCG<br>TATACCGGAGGCGTTGGTCATCAGTGTTGCAACATTTGATTCGTTC<br>TTGAAGAAGACAGCAGCTTTCTCACGGTCCAATCCAGTCATGCAC<br>ATCTCGAGTGTGTACACAGGAATATCAGCTTCGACTGATCCAGCA<br>GATGCAGAATAGATATGCCATTTCTGCTGCTTATCAGAGTCTCCCA<br>TAACATAGGCTTTGCTTCCACTTGAACCAAGCTTGCCAAAATAGC<br>CATCAAGTACGGCCACCTCCTCAGAGAAGTTACGATGAGGGAATG<br>ATTGAGCTCCTGGAAAGATAAAACTTCCTCGTGTGTATCTCACATC<br>CTCAACAGAAAGGGAAAGGAGCTTGGCCAACTTAAGTATGGGAG<br>GGATTGACAGAAGCAATTTAGTTGTTCCACAAGTTTTGATGATAAT<br>CTTGTAGGAGTAAACAAAGAGGCTGGACTCAGAGAGGACATATG<br>AGTCGACATGCTCATTTGACAACGAAGCAACAATGGTGCATTCAG<br>CAGGTTGTAAAATTTCATCTAGTTGGGCTTTTGAAAGAGAACGGA<br>GTCCCTTACCCTCAGGATCAGCAAAGAAGCTTGGTTCAAAAAAC<br>GAGATTTCTAGTCTTTTTTCAAAACCTTCAAACCCAATTGCAGAG<br>ACGTTTTGAGCCATTTTGCAAGAAGAAATTCAGACACCACTAGAG<br>AGCAGAAATGAAGGCGAGATTGAAGAAAAGGAGTTTTGAGTAAA<br>AATAAACTAATCTAATTAACTAAATAAAAGCAGTAAATACGTCAAA<br>TGACGCGATTCCAGAAACAATTAACGAATATCAGGATGGCTTGCG<br>AGCGCAGTT	XM_057909663.1
GAPDH	c118460	GGAAATACTAACCGCACCTATTCACCTCACATTCCACACACACCTT<br>TCTCACTCTTTCTCTCTCTCTCTCTCTCGACTCCTGCTCCTCCACT<br>CCACTTTCCTTTATACTCTACAAACAACAAATGGCCAAGATTAAG<br>ATCGGAATCAACGGTTTTGGTAGAATTGGCCGTCTGGTTGCTAGA<br>GTTGCTCTTCAGAGAGACGATGTTGAGCTCGTTGCTGTCAACGAT<br>CCATTCATCACCACTGACTACATGACCTATATGTTCAAGTATGACA<br>GTGTCCACGGCCAGTGGAAGCACCATGAGCTCAAGGTTAAGGAT<br>GAGAAAACCCTTCTCTTTGGTGAGAAGTCTGTTACTGTTTTTGGC<br>TTTAGAAACCCTGAAGAGATTCCGTGGGCTGAGACTGGTGCAGA<br>ATACATTGTGGAGTCTACAGGTGTTTTCACTGACAAGGATAAGGC<br>TGCAGCCCATCTGAAGGGTGGTGCAAAGAAGGTTATCATTTCTGC	KF234408.1

		TCCAAGTAAGGATGCACCCATGTTTGTTGTTGGTGTCAATGAGAA<br>GTCGTACACGTCAGATCTCAATATTGTTTCTAATGCTAGCTGTACC<br>ACAAACTGCCTTGCTCCATTGGCAAAGGTTATTAACGATAGGTTT<br>GGTATTGTTGAGGGTCTTATGACAACAGTCCACTCTATGACTGCA<br>ACACAAAAGACTGTTGATGGACCATCTTCCAAGGACTGGAGGGG<br>TGGAAGAGCTGCATCATTCAACATCATCCCAAGTAGCACTGGTGC<br>AGCTAAGGCTGTTGGCAAAGTTCTCCCTGCTCTGAATGGAAAATT<br>GACTGGAATGGCATTCCGTGTCCCAACAGTTGATGTTTCTGTGGT<br>TGATCTCACTGTGAGGTTAGAGAAGGAAGCTACTTATGAAGAAAT<br>CAAAGCAGCAATCAAGGAGGAGTCTGAGGGGAAGATGAAGGGA<br>ATCTTAGGCTACACTGAAGATGATGTTGTGTCTACTGATTTTGTTG<br>GTGATAACAGGTCAAGTATCTTTGATGCCAAGGCTGGAATTGCTC<br>TGAGTAAGAATTTTGTCAAGCTTGTTTCGTGGTACGACAACGAAT<br>GGGGTTACAGTACCCGTGTGGTTGATTTGATCAAGCACATTGCCG<br>CCACTAAGTAACTTGGTATGGCTTGCTATAATCATCTAGGGTTCAC<br>ATCTGCTATGGTGGTTATGTGGAATTTGAGTTGGAATAAATTGTTG<br>TCTTTCGAACTAAATACTAGATGTGTGGCTTGTTGTAGCATGCGTT<br>TTCTGGTAGATCTGTGGGATGTTGACTACTCCTATATTCTGAAGAA<br>TTTTGTGTTGAATGGTGAATGTTAGAGTAGTTAATGTGTAGTAGTT<br>TGTAATAAAAGTATTGATTTCTGGCCTTTGCATTGTCGAAGGTGAA<br>ATCCTTGAATAATTGATGGACTGAATGGTTCTTGTTGACCCTTGCG<br>TAGTCCGAGAAGGCATTTTCTAAGAATGGTTTGGCTGTTAGTGTTT<br>GGAGTGATCCAGATATTTGCAAGTTGTTGGTCATTCCAACTTTTTG<br>ATATCCCTTGTGATCTACTTTGTGGATGGTGGTTGGGCGGCAGGCA<br>AGTATCTTTTCATAACTTCTACTCTCTCTGTCTCATCACAGTTGTCT<br>TGTTTTCATTTTCGGGACGTCTCACGCAAGTTGTCCTATTTCCTTT<br>GCAGGAAATAATTTTTGCATTCACACTTGTTTTTACCTTTTTAGATT<br>TGCATTCACACTTGCTTTTTCAATAAAATACCTCACTAAAAACACA<br>TCTACTATTTTTTCTTATTTGACGTGCAAAACACAATTAGGACAAC<br>TGTGGCGAGACGGAGGGAGTTTTATAATTTATAGGTAGGTATAGGT<br>CCACTCATGTGAGTATTAGGTCTGCTTTTTTATGTGAACTGGCAAG<br>TGGGGCACAGAGCCGTTGTTTTTGGTTTTACTTGTGCAGTTTTTGT<br>ATTCCAAGTCCCTATTCAATAGATATGTTACATTTGAGAATATTTCA<br>ACACCTCGGAGATGTTATTGAGGGAATAGATAGAGATTGGGGTGA<br>ATAGGATTGATTTCAATGGCAATTTTGCAGTCATTTTCTCCTTTCAC<br>AGCCCATTTACTTATACCTGTCCTATCTTTTACGTCTTTTTTTTTTT<br>TTTTTA	
eIF4α	c118665	GAGAAAATCAATTTAAAGTGTTAAACCAACATGTCGATTAAAATG<br>TAAATGGAATAATTTAAGAGATTATTAGATAAAAGTTAATATAAATA<br>TTCAAAGCTGAAAACAAAATCTATATTTGCAAACATTGTTGATTGT<br>TCGCATATCTCTCTCTCTCTAAGTTGGAAGAAAATCAAGAAATGG<br>ATGCTTCTGAATCTCCTCAGTCCACTCCTCCCTCTCCGCCCTCCAC<br>TCTAAGTACACAACGCCATTTCTACCTTGCTGTTGACAGACTCCA<br>ATTCAAGATGGAAACGCTGTTAGGTCTTCTAGGCATGGCTGGGAA	XR_006052213.1

GCGTCCTTGTCTTCCTATGGTCGTGTGTTGCAGCACTCGCGACGA
GCTCGACGCCGTCTGCTCCAATCTATCTACTCTATCGTCTTATGTTT
CTGTTTCCCCACTATACAGCGACCTTTCCGAAGTCGACAGAGCGG
AGGTTATGGATAAGTTTAGGCAGGCCACTGCGAGATGGAATAGGT
TGAGAAGAGGGGAGGAAGCTGAGAGTGAGGCAGATGAGGAGCG
GAAATCTCATGTTGTTGTCGTTACTGATGCCTGCCTTCCGCTTCTC
GGATCTGTTGAGTTGCCTCTTTGTGCTTGCATGTTGATCAACTATG
AACTGCCAACAAAGAAGGAGACTTATGTGAGACGCATGACAGCT
TGTTTGTCGCCAGGTTTGTTTTAAATCAAAGTAGTGTGAAAATTTT
CCTTGCTATAATTCTGAGCCCCCAACTAAATGTTCTTTTGTCCTAAT
TTTCCACTTCTAAGACATGCAAAATTGCTGAATAGTACTTTTATATG
TCAGCATTTCCCCTTTCCATTGTATCATGTTTTGCAATTTCCTCTTA
TATTGAAATGATATTGGCATTGTTATTTCCTGAGCATAAAGATGGA
ATAGTGATTAGCATGGTTGTTGGAGGAGAAGTAGTGATTCTTAAA
AACCTCGAAGAGAACAACGGTCTTGTCATAGCTGAAATGCCTATA
AATATCTTTGAGATGATTTGAGAATACCAACCCTCTTGGAGTGCCT
GAAGAAAATGTAAGGTGTAACGCACATACCTTCTCAAGTGGTATT
TCTTATAGTTTATACTGCTTTTTGGCATGTGGTGTCATGTTTCATAC
AATGTATGAACATAATTCTGATTGCATATGTATTTAATATTTTTGTAG
CAATAATATAACCTTGTATGTATCTATTGTGAGAAATATAATGCTTG
CATCCCCAAGTTTTGAATGTACTTGGCAGGAGTCTTGTCAATTTTT
TCTCTCGTAGATTTTCAGTTCTTTCCTACCTTCAAGAAGTAGGGGT
GTTTCAGAGAAAGACTGAAAAGCTTGGATGGAACCTTTTCTTTTT
GGGATAAATGGTAGAACTTAACGAGATATTATGCGCAACTTAGCG
AAGGCATAGCTAACAGTGAATGCTCCAGATACGCTAGAAACATAA
GAAGAAGAAAAGGAAAAATAAAAAATAAAAAATAAAGTGGGGA
GGAACCAATACAGGTCTTGATAGTTTTGTAATTGATGGTCTGCTAG
AATCATTAATATCTGGCATCTTTCTTGAATTTTAGGATTTGTTTGGA
TTGCTGAATTTTAGTTTTTGTCTGTTTCTATTATACGCATGCACTGG
ATTTGGGGATACTCTACTAGGACCTAGATAATAGAATTTCGTTAGT
TAATATTCATTACATCAAAAACCTTCAGTTTTTCTTACGACTTTGA
AAGGAAAATGAGAAATGGAAAGAAAATGATAGCAGCGGGAATGA
TTTTGTTTTCCTGACATTGAGGGCTATAACACATTGCCTAAACCGA
AATATCAAAATGCTATCCATGAGATATTGAGATCATACCTTCTACCC
AGCATCACATGTCTATTCCTTTCACCAGTCCCTCATATCCAAACTT
TGTGTTACATGAACTTGAGAGTATGGTCTCAATTGTAAACATATCT
TGAGTTTTGACTTTGATATATTTGTCCTTGATTCTTGTAGAAATATA
AACCACTGCACATGTCAATATACTAAGAGGTCTTTCCTCCCTTTAT
CTGTTTGGCATGCAAGCCAAAAGATCTGGAGGTGCACTTTTGGCC
AGCAAAGCAAGTAAATTGGTTCATTTGTAGTTGTACTTTGCACTTT
GCTGGTATCTCACGTCCAACAAATTCAGACTCTGCACTAGTCTAC
ATACTGTGGCTAGCATTTTCTCACGTCCAACAAATTCGTGATGCTC
TTTCTTGATCTGAATAATTTATTTTTCTTTTGTCATGAACAACTTCA
TATCTGAAAATAGGCAACTTTTTTCCCTGAAATTTCCAATTTCTAG

		AGTCTTGATACCATTTACATTGCTGCCAAAGAATGGCCCATGCTTC<br>ATATGAGTTGTTTCTGCATCATATGAAGGGATGGTAGGGGACCAG<br>GATTTATTTTAGATTTTTAGGACAGAAGTTGATTATGTAATGCTTTT<br>AGGGTGAAACTTTTAATAGGCCCACTTCGAGTCCCGTTGTTTGAA<br>TGTGTTGCATGTTCTTCAACAGGAAATTAGGTCCAGCTCTAGGTC<br>CCCACAGATGGATGAAATGTTGCTTTTCTGAAGCTCATATAGGGAT<br>TATGAGTAATATTTTCCAGTTGCTTATGGCTTTAGGTCACTCATATT<br>TTATCCATTTAGGGTAGATAATTTATCTCCTCGGCATTGTGGTTCTG<br>GCTCAGTGATTTTTTAGGTGGCTTGTGTCTGAATCGCATCCACTAG<br>ATAATTGGCAGGTGCTTTAAATGATTACATAGTAGTGGAAGATGTT<br>TTAGTTGGTTTCTAAATTTAGAAATATGAGGTAGTAAACTCATTTTT<br>ATATAAAGTAAACTATGTTGAAATCCGTCATTTTCGTGTATATTATT<br>TGAGTAATTTTGACTTGCTTCTCTTAGCAAACTACGTTTATTTGTCT<br>CCTTTTGGCTAAGCCTTTCGATACTTTAGACAGCATAGTACAAGCT<br>GTATCTACTGACTTTTTCTGGAGATCCTTGAGAATATGGTTTACAC<br>TAATTACACCAGTCTCCATATTGGAACAATGAAATTATGCCTTTAAT<br>TCAGAAGTATGGGTTGCTGATACTGTTGCTTTCTGCAATACTTTTC<br>TCTCAACTGCAGTAACTCTTATTTTTCATTTGCAGTGAAGATGAAA<br>GTCGCAATATAAGTTTAGGCAGGGTGACAACTGACAATTGCTAGA<br>TCCTTTGCTACGTTAAGAATTCCAAAGCACAACACATGATCTCTTC<br>TCATGGTGGTATTTCCTTTCCAGTTTCTTCTGTTCAATAGTATGTTA<br>GTCCATTTCTTACTGCACGGTTTGAAAATATTTTTCCAATTCAACG<br>ACTCTCAAAGTTTGTCATCTAAAATAGAACAACATCAACACTCAA<br>CAGTCTTTCTTTTGTTGCTCAGCTGTCAACATGAAATTATCTTATTG<br>CTAATGCTTTCTGAGTTGCACAGAAAGATGCGGCAAAGAATAAAT<br>GAGATTCCCCTTCTTTCCAAGATCATGCCCAAATTAGAAAAGTTG<br>GTATCTTCTTAAATTGATGGGTTAATCACTAGAATCAATTGGGGTAT<br>AAAATTGACAGTTGAACAACTATAGTTTTTGTAGTAACGATACTTG<br>ATGTCAAAATCGTCCTTGCTTTTTACCCTTTTGGAGCAATGACACC<br>CCAGAAGTGGGGGTATGAGAAAGCAAGATTTTGTCTGTCTTAAGG<br>GAAGAAGGAGAGATTAATGACACATGGTCCCTTTATTTTTTATTTA<br>TTTTTCCTTTTCTTGTGATGATATTTGCTAATTATATTTGTTTTAATTT<br>CTTTATAATGCAATGATACATCCCTTTTTGTGGCCTACCCCTCTGCC<br>TTTACCCCTCTGACTCGCTGTGCTGTATCGAAAGGAATGCATTATA<br>AAAGCAGT	
TUB	c117718	CATGCATCATCATCCCTATATGAACAACTGAAGTTACAATGCAATG<br>AAATTGTACGCGAATTAATACGGAATAGTGAAAAGAGTTGACATC<br>CTTTTGAATTAATACGGAATAGTGGAAGAGTTTGACAACCTTTTTC<br>CTCAAAATATGTACTCAAAAACCTCAAGAAAGATAATGCCTGGAA<br>CTTGAAAGATTACAACACAAATGCAACTGAAAAGGTATTTGAGG<br>GCTGAGTAACTGAGTTGATCATTTTATATGATACAGCTTTCAATATT<br>CTTCTCCTTCCTCTTCCTCTTCCACATCTTCCGAAGCCACTTCTTC<br>GTAGTCCCTTTCAAGAGCAGCCAGATCCTCGCGTGCTTCAGAGAA<br>TTCACCTTCCTCCATCCCTTCACCAACGTACCAGTGAACAAATGC	XM_057912730.1

TCGCTTTGAATACATCAGATCAAATTTGTGGTTGATTCGAGCAAAA
ACTTCAGCCACGGCTGTATTGTTGCTAATCATGCACACTGCACGTT
TCACCTGTGCAAGATCACCCCCTGGCACCACTGTCGGAGGCTGGT
AATTGATACCACATTTGAAACCAGTTGGGCACCTGCAAGTACGGT
CATGTCATGTCACTAACAGCACGAACCAATCAAATTTACCAAAAA
TCACCCACCACTCCCTTGAGGTGCAAACACAAATAAAATTGGTTT
ATTTTCTAGTGGAGTCCCATAAACCTACACCAAGGAGCTCTCAAA
CTGGATAAACATGGGTCTGCAGTAGGAAACTTAAGATATTAATATA
AAGCAGGAGTAACTGAAGCCAATAAACTCATATTCCATAAAGCTC
TATTGACTTTTGAGGAATCCATCACTAGATCTCCGTCATGTAATCA
AACAAAAAACAAATTATTCATTTGATGTCTTCCTATTTTGTTATAGT
CATATATTTTACTGGAGAAGACAACTTAATATTATATTCCGGGAGCT
AGATAGAGGAAGATTACCAGTCGACAAACTGAATAGTCCTCTTAG
TTTTGATAGAAGCAACAGCAGCATTAACATCTTTTGGGACAACAT
CACCACGATACATCAAGCAGCATGCCATGTACTTCCCATGCCGTG
GATCACATTTTGCCATCATACTTGAAGGTTCAAATACTGCATTTGT
GATCTCTGGAACAGATATCTGCTCATGGTAAGCTTTAGCTGCTGAG
ATAACTGGTGCATAAGAGGAAAGCATGAAATGAATACGAGGATAA
GGAACAAGGTTTGTTTGGAACTCAGTGATGTCCACATTAATGGCG
CCATCGAATCTCAAGGAAGTGGTCAAGGATGAGATTATTTGTGAA
ACCAAGCGGTTCAAGTTTGAATAAGACGGCCTCTCAATGTCCAAT
GATCTCCTGCAAATATCATATATAGCTTCATTGTCCAAAAGAACCA
CAACATCTGTATGTTCTAGAAGGGAGTGAGTGGACAAAACACTGT
TATAAGGCTCTACCACAGCAGTCGAAACCTGGGGAGATGGGAATA
TACTAAAGCCAAGTTTCGACTTTTTTCCGTAATCTACAGACAGAC
GCTCAAGAAGCAGGGACCCGAGGCCAGAGCCAGTGCCACCACC
AACAGCATTGAACACCAAAAATCCCTGCAATCCAGTGCAGTTGTC
TGCCAGCTTCCGGACTCGATCAAGGCATAGGTCAATGATATCTTTG
CCAACAGTGTAATGTCCTCTAGCAAAGTTATTTGCCGCATCCTCCT
TTCCAGAAATGAGTTGCTCAGGATGAAAGAGTTGCCTATATGTCC
CAGTCCTAACTTCATCGATAACAGTGGGTTCAAGATCGACAAAAA
TAGCACGAGGCACGTGCTTGCCACCGCGGGTTTCGCTGAAGAAG
GTGTTGAAAGAGTCGTTTGCAACTCCAACAGATGTATCGCTAGGC
ATCATTCCATCGGGCTGAATGCCGTGTTCGAGGCAATAAAGCTCC
CAG

Table S2. Primer sequences and amplification characteristics of 12 candidate reference genes for RT-qPCR analysis in *Dracocephalum moldavica* L.

Genes	Forward primer (5'-3')	Reverse primer (5'-3')	Product length (bp)
18S rRNA	CACCGCCACGGACAGTCTC	CAACCCGCATTTCTTTTAACAA	199
ACTIN	CGATCATTGATGGCTGGAAGAGGACCT	GCTCTGCTGTGGAGAAGAACTACGAACT	105
HIS4	AAGGGAGGCAAGGGATTGGGAAAGG	ATCTCAGCCGCCGAAACCGTAGAG	300
HSP70	TTCAGTTCTTGAGGTCGGAGATGGAGTT	TTCTCAGCAGATTCAGTAAGCCGTTGGA	186
28S rRNA	TTACAACATCTGCCTCCTCCATAACCTCCT	AGGTCGGCTTACTTCGCACGGAGA	208
ARF	GCGTTGTTCTCAGGCAGCTCCACAT	CTTACCAGCAGCATCAAGACCAACCATCA	205
CAC	GGGAGAAGACAACTAGGCAAAGTACAAGGA	CGCTGTGAACAACACGGACTCATTACC	150
EF1α	CTTCACCATACCAGCATCTCCGTTCTTCAG	GCCAACTTCACTTCCCAGGTCATCATCAT	198
SAMDC	CTTCTTCCTCGTCCTCATCATCGTCTCTG	GCTGTGAATCTGCGTGCTCTGGTC	274
GAPDH	AAGCAGCAATCAAGGAGGAGTCTGAGG	CATACCAAGTTACTTAGTGGCGGCAATGTG	242
eIF4α	CACTGCGAGATGGAATAGGTTGAGAAGAGG	AGCGGAAGGCAGGCATCAGTAACG	105
TUB	CTTCTCCTTCCTCTTCCTCTTCCACATCTT	GGTATCAATTACCAGCCTCCGACAGTG	283

Table S3. Primers sequences of *DREB* and *NCED*

**Genes**		**Primers (5’-3’)**
DREB	Forward	TCAGTCGAGCACAAGGACCATACA
	Reverse	TTACAGAGGCGTGAGGCAGAGG
NCED	Forward	GTGCTCGCCGTAGATGAACTTG
	Reverse	GCCGACACCTTCTGCTTCCA

Table S4. The counted and ranked the top five genes by five different programs

Samples	Ranking	ΔCt	geNorm	NormFinder	BestKeeper	RefFinder
PEG treatment of leaf	1	*ACTIN*	*EF1α*	*SAMDC*	*ACTIN*	*ACTIN*
	2	*GAPDH*	*GAPDH*	*HIS4*	*GAPDH*	*EF1α*
	3	*18S rRNA*	*eIF4α*	*18S rRNA*	*18S rRNA*	*18SrRNA*
	4	*EF1α*	*28S rRNA*	*ACTIN*	*EF1α*	*GAPDH*
	5	*eIF4α*	*ARF*	*GAPDH*	*eIF4α*	*eIF4α*
PEG treatment of root	1	*GAPDH*	*EF1α*	*GAPDH*	*EF1α*	*EF1α*
	2	*ARF*	*SAMDC*	*ARF*	*ACTIN*	*eIF4α*
	3	*EF1α*	*18S rRNA*	*ACTIN*	*18S rRNA*	*GAPDH*
	4	*eIF4α*	*GAPDH*	*EF1α*	*CAC*	*ARF*
	5	*18S rRNA*	*ARF*	*SAMDC*	*GAPDH*	*ACTIN*
Flower stages	1	*28S rRNA*	*18S rRNA*	*HSP70*	*28S rRNA*	*HSP70*
	2	*18S rRNA*	*HSP70*	*CAC*	*18S rRNA*	*SAMDC*
	3	*HSP70*	*ACTIN*	*SAMDC*	*HSP70*	*ACTIN*
	4	*ACTIN*	*EF1α*	*ARF*	*ACTIN*	*EF1α*
	5	*CAC*	*SAMDC*	*ACTIN*	*CAC*	*CAC*
Different tissues	1	*eIF4α*	*GAPDH*	*ARF*	*18S rRNA*	*ARF*
	2	*18S rRNA*	*ACTIN*	*28S rRNA*	*eIF4α*	*EF1α*
	3	*28S rRNA*	*EF1α*	*ACTIN*	*ARF*	*ACTIN*
	4	*ARF*	*ARF*	*SAMDC*	*28S rRNA*	*28S rRNA*
	5	*SAMDC*	*SAMDC*	*GAPDH*	*SAMDC*	*18S rRNA*
Total samples	1	*18S rRNA*	*EF1α*	*ARF*	*18S rRNA*	*ARF*
	2	*eIF4α*	*ACTIN*	*SAMDC*	*eIF4α*	*ACTIN*
	3	*SAMDC*	*GAPDH*	*ACTIN*	*ARF*	*EF1α*
	4	*ARF*	*SAMDC*	*GAPDH*	*28S rRNA*	*18SrRNA*
	5	*28S rRNA*	*ARF*	*28S rRNA*	*SAMDC*	*SAMDC*

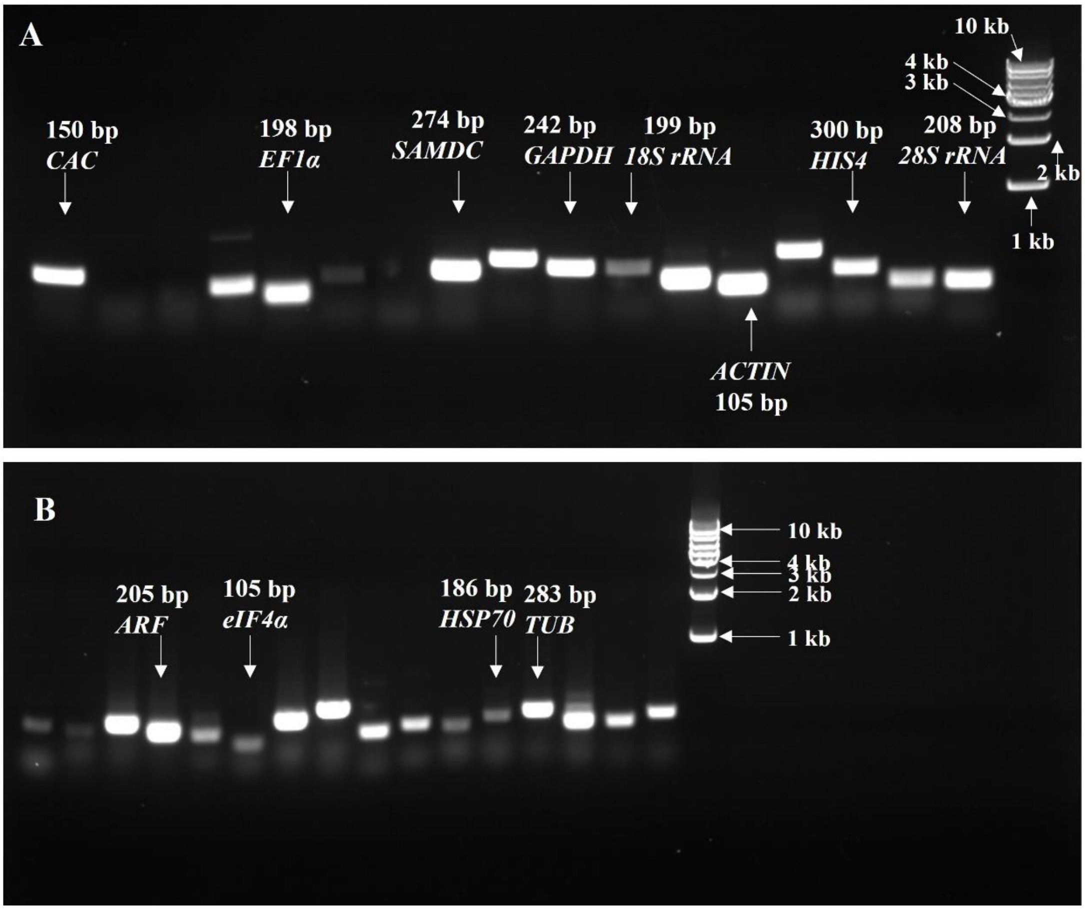

Figure S1. The primers' specificity of 12 candidate reference genes detected by agarose gel electrophoresis in *D. moldavica*.

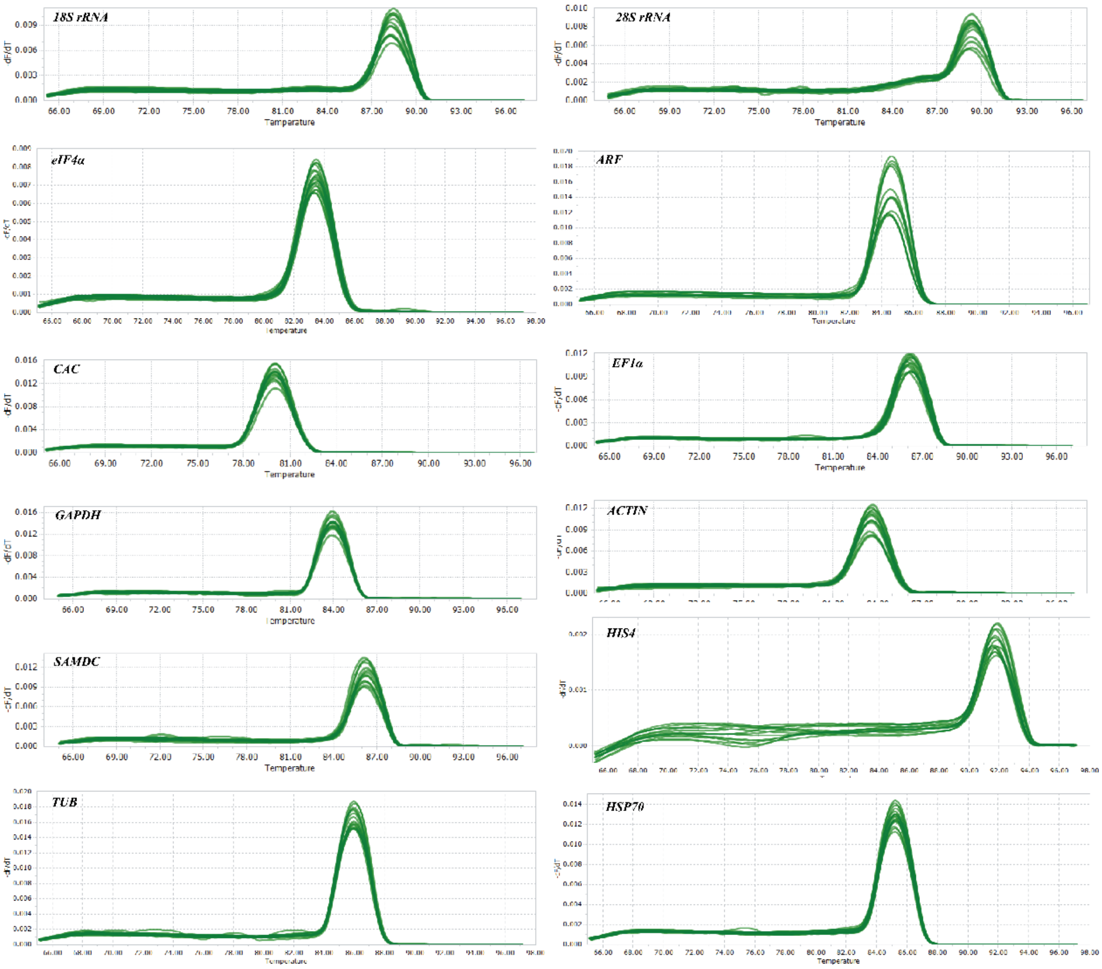

Figure S2. Melting-curve analysis of quantitative real-time PCR (RT-qPCR) amplification of 12 candidate reference genes in *D. moldavica*.
